# Heterogeneous quantization regularizes spiking neural network activity

**DOI:** 10.1038/s41598-025-96223-z

**Published:** 2025-04-23

**Authors:** Roy Moyal, Kyrus R. Mama, Matthew Einhorn, Ayon Borthakur, Thomas A. Cleland

**Affiliations:** 1 Computational Physiology Lab, Department of Psychology, Cornell University, Ithaca, NY 14853 USA; 2 AI for Science Institute, Cornell University, Ithaca, NY 14853 USA; 3 Neurosciences Graduate Program, Stanford University, Stanford, CA 94305 USA; 4 Mehta Family School of Data Science and Artificial Intelligence, IIT Guwahati, Guwahati, Assam 781039 India

**Keywords:** Neuromorphic, Artificial olfaction, Preprocessing, Signal conditioning, Representation learning, Network models, Sensory processing, Computational science

## Abstract

The learning and recognition of object features from unregulated input has been a longstanding challenge for artificial intelligence systems. Brains, on the other hand, are adept at learning stable sensory representations given noisy observations, a capacity mediated by a cascade of signal conditioning steps informed by domain knowledge. The olfactory system, in particular, solves a source separation and denoising problem compounded by concentration variability, environmental interference, and unpredictably correlated sensor affinities using a plastic network that requires statistically well-behaved input. We present a data-blind neuromorphic signal conditioning strategy, based on the biological system architecture, that normalizes and quantizes analog data into spike-phase representations, thereby transforming uncontrolled sensory input into a regular form with minimal information loss. Normalized input is delivered to a column of spiking principal neurons via heterogeneous synaptic weights; this gain diversification strategy regularizes neuronal utilization, yoking total activity to the network’s operating range and rendering internal representations robust to uncontrolled open-set stimulus variance. To dynamically optimize resource utilization while balancing activity regularization and resolution, we supplement this mechanism with a data-aware calibration strategy in which the range and density of the quantization weights adapt to accumulated input statistics.

## Introduction

Learning relevant features from unpredictable input distributions is a critical task for any neural network architecture. Under realistic conditions (“in the wild”^1^), robust representation learning hinges on the quality of signal preprocessing, which in turn depends on the successful application of domain knowledge. Biological sensory systems solve such ill-posed problems by embedding the relevant domain expertise in the functional architecture of their neural circuitry, utilizing a range of computational tools including specialized neuron types, synaptic connectivity patterns and weight distributions, and local plasticity rules^2,3^ that have evolved to solve modality-specific challenges. Neuromorphic computing strategies incorporate similar biomimetic principles^4^, defining the network architecture as part of the algorithm and favoring efficiency and robustness over generality. What, then, is required of a signal conditioning mechanism in a neuromorphic artificial olfactory system intended for deployment in the wild?

Unregulated chemical signals (that is, volatile or solute molecules, referred to as *odorants* or *analytes*) are challenging to sample and interpret reliably. Under natural conditions, they are prone to unpredictable source mixing and environmental interference; moreover, they often exhibit intensity ranges spanning several orders of magnitude, with potentially relevant information embedded across multiple scales. The chemosensory modality also lacks intrinsic structure analogous to spatial proximity in visual scenes or tone frequency in auditory samples. Whereas the basis for chemosensory similarity is mediated by overlap in the distributions of activated primary sensors, as it is in these other modalities, the neighborhood relationships among their receptive fields are not fixed with respect to physical parameters such as wavelength, proximity, or frequency. Rather, in biological systems, they are determined by pairwise binding affinity and efficacy profiles exhibited between odorant ligands and primary chemosensory receptors. High perceptual selectivity is achieved by deploying large numbers (100 s to 1000 s) of receptors exhibiting diverse chemoreceptive fields, such that the activity pattern generated by a given odor scene^5^ is diagnostic of the analyte(s) present, with enough redundancy to enable denoising and source separation. The selectivities of primary chemosensors, the numbers of different chemosensors deployed, the diversity in the pattern of overlaps among their receptive fields (within a given environmental context), and the limitations of their transduction mechanisms impose fundamental bounds on the range, resolution, and discrimination capacity of the resulting chemosensory system^6^.

Many artificial chemosensory systems are biomimetic, deriving overall selectivity based on arrays of partially selective primary chemosensors^7^. Indeed, the properties of contemporary sensor technologies are a limiting factor in artificial system performance. The most well-established technology relies upon arrays of inexpensive metal oxide gas sensors, which are limited in their potential by both lower selectivity (broader receptive fields) and low diversity. Conducting polymer sensors enable increased diversity, though the adsorption-based transduction mechanisms of both of these (and many other^7^) chemosensor types render them selective only for whole-molecule properties, rather than the “odotope” (molecular epitope)-based selectivity of biological receptors^5,6^. Sensor arrays based on peptide aptamers recapture some of the capacity for odotopic selectivity while retaining the capacity for traditional modes of deployment^8,9^. They also can exhibit substantial receptive field diversity based on different peptide sequences, and can be designed for specific selectivities via phage display^10,11^. Engineered chemosensory systems based on complete biological receptors^12^ enjoy the selectivity and diversity advantages of biological systems, but require specialized and sensitive deployment mechanisms. Finally, chromatographic separation followed by mass spectrometry enables the identification of molecular species by mass, and advances in tandem mass spectrometry enable some additional selectivity beyond whole-molecule mass^13^. However, the analytical process is slow and laborious.

Various strategies have been deployed to identify specific chemical analytes with artificial chemosensory systems. One strategy is simply to attempt to narrow the selectivity of a single sensor so that it detects only the analyte of interest. This can be achieved directly for certain chemically distinctive analytes (e.g., oxygen, carbon monoxide), for which such sensors are already commercially available. Alternatively, the deployment environment can be controlled such that a less specific chemosensor is only ever exposed to one analyte to which it is sensitive, thereby rendering the sensor highly selective within the context of that highly regulated environment. This is how synaptic neurotransmitter specificity is achieved within biological brains^6^, but is an ineffective strategy in the wild. A third strategy, utilized in biological olfactory systems as well as in biomimetic artificial systems, is to deploy arrays of chemical sensors, each of which may interact with multiple analytes, and achieve specificity by interpreting the patterns of partially-independent responses across the elements of the sensor array^6,14,15^. In these *multivariable sensors*, analyte response patterns can be correlated across analytes or channels, owing to similarities in analyte molecular structure or physicochemical properties (depending on the physics of sensor transduction); it is these correlation patterns from which specificity is ascertained. However, these correlations are highly vulnerable to the source mixing, interference, and scale problems presented by unregulated sensory environments. Moreover, chemical analytes can be encountered at operationally relevant concentrations ranging from a few parts per billion up to overwhelming intensities that saturate the responses of primary sensors. The sensitivities of different chemosensors to particular elements of a chemical environment can vary by orders of magnitude, and their responses to increasing concentrations can be sharply nonlinear. Different types of chemosensors are vulnerable to different subsets of these problems^7^. Accordingly, a wide variety of post-sampling strategies for analyte recognition, primarily based on classical machine learning methods, have been used to address the challenges presented by multivariable chemosensory data^16,17^.

**Fig. 1:**
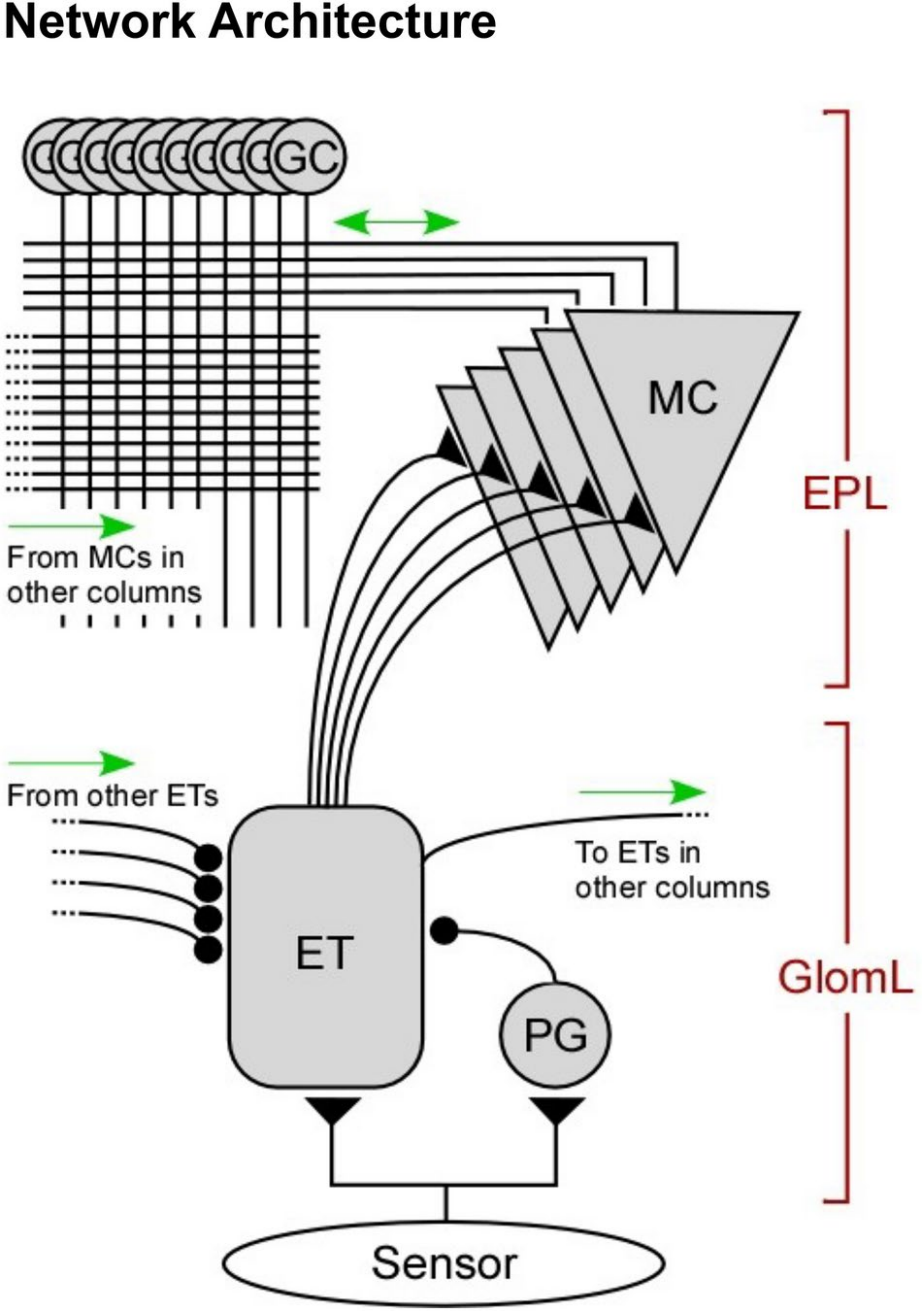
Neuromorphic network diagram of a single sensor-associated column, directly derived from the circuit architecture of one column of the mammalian olfactory bulb (OB). Input from a specific sensor class is delivered to external tufted (ET) neurons in the first layer, based on the glomerular layer (GlomL) of the olfactory bulb. In the artificial network, ET cells are non-spiking and deliver graded feedforward synaptic excitation onto mitral cell (MC) principal neurons, generally with heterogeneous synaptic weights. ET cells also inhibit one another across columns in a nonspecific mutually inhibitory network that subsumes the role of inhibitory ET cell-driven periglomerular (PGe) and superficial short axon (sSA) cells in the biological network^21,23^; this network effects global normalization across the sensory input field. MCs are spiking neurons that initiate dynamical, spike timing-dependent interactions with inhibitory granule cells in a plastic network modeled after the external plexiform layer (EPL) of olfactory bulb^18^. The number of MCs per column was systematically varied between 1 and 32, whereas the number of GCs was always four times the number of MCs (see *Methods*). Spiking MCs excite GCs with a fixed probability across the full network, irrespective of column, whereas GCs inhibit MCs only within their home column^21^. In the present study, GC → MC inhibition was disabled to isolate the effects of the quantization step (ET → MC). Filled triangles depict excitatory synapses; filled circles depict inhibitory synapses.

We have previously presented a neuromorphic algorithm, based on the architecture and function of the mammalian olfactory bulb external plexiform layer (*EPL*; Fig. 1) and implemented on Intel Loihi. This method learns multiple arbitrary representations rapidly and sequentially, without catastrophic forgetting, and subsequently can identify them even in the presence of competitive background interference^18^. Specifically, that EPL network is robust to impulse noise affecting a subset of its sensors and to a limited amount of variance across all sensors, but it relies on input statistics that are fundamentally well-behaved. Hence, transforming the uncontrolled variance of the external world into a sufficiently regularized, yet still informative, activity distribution must occur prior to EPL activation. In the biological olfactory system, there are multiple mechanisms described that preserve representational integrity across wide analyte concentration ranges^19^ and compress it into the limited dynamical range of olfactory bulb circuitry^20^. It also is established that diffuse lateral shunting inhibition in the olfactory bulb glomerular layer (*GlomL*; Fig. 1) delivers divisive global normalization across the array of OB *columns*, each of which is associated with one of its hundreds of different receptor types. This enables concentration invariance in downstream areas by ensuring that the total activity across OB columns is stable across input samples^21–24^. However, we here show that this normalization, while necessary, is not sufficient to provide the EPL with statistically well-behaved input.

To resolve this problem, we present a regularization mechanism predicated on signal duplication combined with quantization weight heterogeneity within columns of spiking neurons (*gain diversification*). We test this mechanism on a feedforward spiking neural network (SNN) utilizing a spike-phase code and local, spike-timing-dependent learning^25^, modeled after the mammalian OB and incorporating new circuit elements drawn from the OB glomerular layer. Specifically, raw chemosensor data are first routed into GlomL circuitry (Fig. 1), which normalizes and regularizes the activity distribution before delivering it to the EPL. Following GlomL normalization^26^, the analog responses of external tufted (ET) cells in the GlomL are transduced into the temporally patterned spiking activity of mitral cell principal neurons (MCs) in the EPL (Fig. 1). By this method, unpredictable and unregulated sensory input can be regularized for effective further processing by downstream spiking layers, without foreknowledge or the need to operationally tune hyperparameters. We describe and optimize this approach (see *Methods*) with respect to multivariable sensor activity regularization, information retention, and resource utilization.

## Results

### Dataset properties and signal conditioning

The response signatures of chemosensor arrays to analytes of interest and their environmental backgrounds are often not statistically well-behaved. High quality response signatures, such as those provided by the biological olfactory epithelium, are high-dimensional, providing diversity across sensors both (1) in the levels of activation exhibited (i.e., some strongly activated, some weakly or not at all) and (2) in the *channel coding* sense, in which different analytes activate correspondingly different complements of sensors. Well-behaved inputs exhibit the former diversity in a consistent distribution, irrespective of which specific sensors are strongly or weakly activated.

**Fig. 2:**
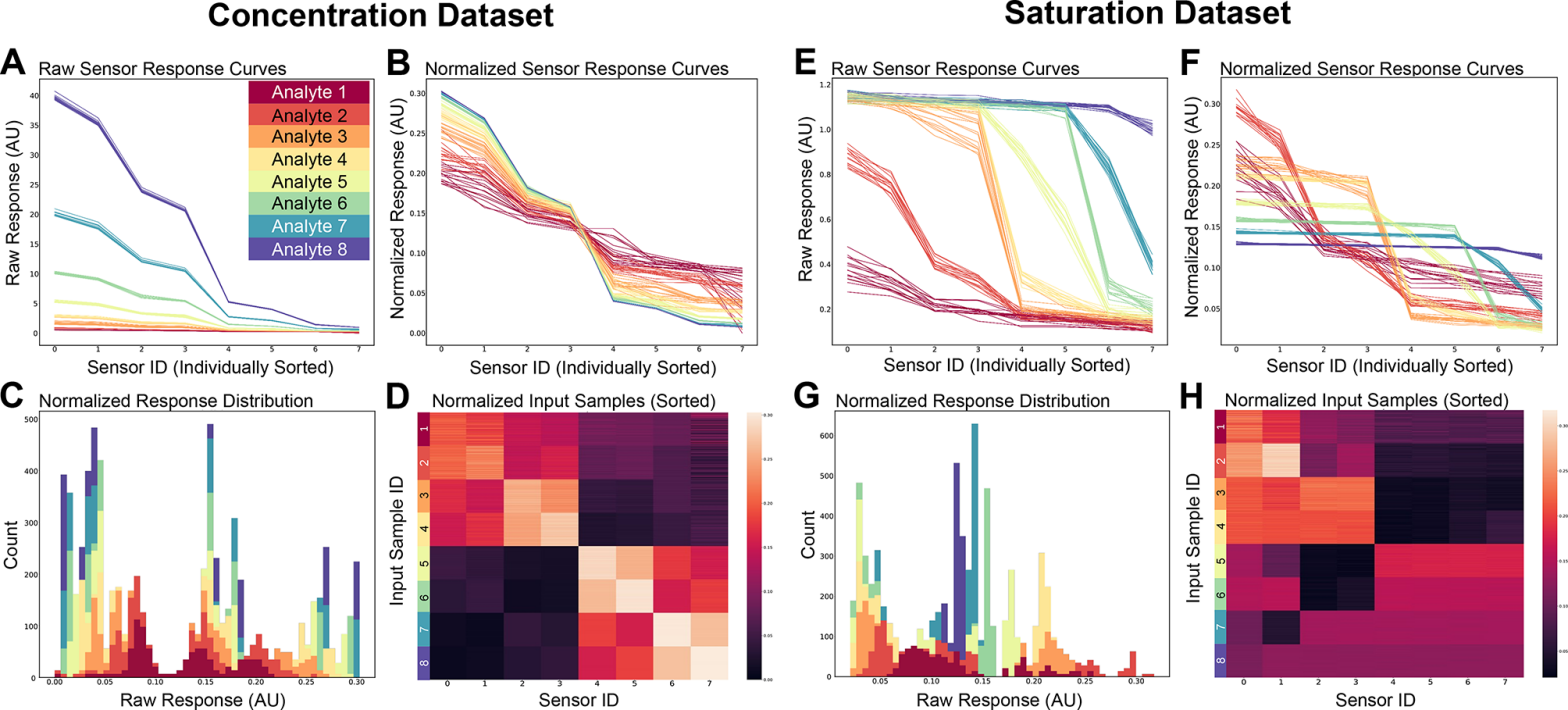
Raw and normalized synthetic sensor responses to test samples from two generated datasets, designed to simulate high variance in concentration or sensor sensitivities (left) and strong saturation effects (right). Responses are aggregated over eight cross validation folds (train-test splits) and color-coded by analyte class. (**A**) Raw sensory dataset with balanced sensor affinities and systematic differences in synthetic analyte concentration. Response curves are sorted individually from most to least active, to emphasize differences in slope (see Fig. S1 for unsorted data). *Abscissa*: the eight individual sensors. (**B**) Dataset from A following global normalization. (**C**) Stacked histograms depicting normalized synthetic sensor response distributions (binned counts), with raw responses sorted from minimum to maximum sensor activation levels. *AU*: arbitrary units. (**D**) Normalized sensor responses in matrix form, sorted row-wise by analyte class to emphasize similarity structure, with sensors on the horizontal axis and samples on the vertical axis. (**E–H**) A sigmoid transformed variant of the first dataset, yielding imbalanced response magnitudes and systematic differences in sensor saturation across analytes. In both datasets, a small amount of Gaussian noise was added to render the classification problem more difficult (see *Supplementary Methods*).

Here, we sought to utilize GlomL-inspired preprocessing circuitry to transform a wide variety of raw input signatures into a common, well-behaved spike phase distribution with minimal information loss. To that end, we generated two synthetic datasets, each comprising eight analytes and activating an array of eight artificial chemosensors. In the first, the *concentration* dataset (Fig. 2A-D, S1), sensor receptive fields were balanced, but were distinguished by different overall sensor response levels—possibly as a result of varying analyte concentrations, or else corresponding to a range of analytes among which that sensor array was highly sensitive to some but not others. In the second, the *saturation* dataset (Fig. 2E-H, S1), the analytes were poorly encoded by the primary sensor transduction process, with some or most of the sensors saturated by analyte presentation, and with relatively few sensors exhibiting intermediate activation values that are sensitive to small differences in analyte quality. Both of these problems are exacerbated by the limitations of contemporary artificial chemosensors. Because we here emphasize the problem of *data regularization*—i.e., transforming input distributions to accommodate the operating range of a downstream spiking network—we depict activity distributions sorted along the abscissa by amplitude for clarity, de-emphasizing the channel coding differences among analytes upon which their classification primarily depends (unsorted activation profiles are depicted in Fig. S1 and in matrix colormap form in Fig. 2D, 2H). To render the problem more difficult, different samples of the same artificial analyte were jittered by adding a small amount of Gaussian noise (see *Methods*).

Raw sensor outputs were each delivered to a corresponding analog neuron modeled after the external tufted (ET) cells of the olfactory bulb (Fig. 1). Initial normalization was performed for each of the eight analytes presented by globally scaling all ET cell activation levels such that their activity summed to a constant (Fig. 2B, 2F). This corresponds to the biological architecture, in which ET cells interact via inhibitory interneurons in a broad, nonspecific lateral network, thereby normalizing chemosensory input into a relational representation^20,22,23,26^. In a neuromorphic instantiation, unconstrained by Dale’s principle, the ET cells simply inhibit one another directly in an all-to-all lateral network while simultaneously exciting MC principal neurons within their own column, as depicted in Fig. 1. Notably, even after normalization, the distribution of activity levels across the eight ET cells differed as a function of the analyte presented (Fig. 2C, 2G); for example, analyte 1 from the *concentration* dataset evoked a narrow range of moderate activation levels across columns, compared to analyte 8 which evoked close to zero activity in some ET cells and near-maximal activity in others. That is, normalization alone does not suffice to regularize sensory input into a well-behaved distribution.

The normalized representations generated in the analog ET cells activated groups of MC principal neurons within the same column (Fig. 1). MCs were implemented as spiking neurons, but their activation levels are represented by spike phase rather than by mean spike rates. Specifically, a sinusoidal background rhythm was applied (known as the *gamma oscillation* after its biological equivalent), MC spike rates were constrained to zero or one spikes per gamma cycle, and the activation level of a given MC was represented by the relative phase of its spike with respect to gamma. This phase coding enables a single spike to embed a numerical value of arbitrary precision, works naturally with established online learning rules such as spike time-dependent plasticity (STDP)^18,25^, and underlies a previously described iterative signal restoration process in a neuromorphic EPL circuit^18^. Here, we establish a fixed precision of 50 discrete phases per cycle; this temporal resolution can be adjusted as required, potentially improving precision or memory capacity at the cost of higher energy-to-solution. The synchronization of MC spikes also governs the recruitment of granule cell interneurons into the activated ensemble (GCs; Fig. 1), as previously described^18^. Briefly, activation of GCs requires coincident spikes from multiple presynaptic MCs. During model training (odor learning), an asymmetric STDP rule tunes the receptive fields of GCs to become selective for input from particular sets of activated MCs, such that individual GCs com to represent specific higher-order features of trained analytes^18,25^. The coordinated tuning of MC-GC–MC synapses is a core component of representation learning in a fully operational EPL model. However, in this study, GC → MC recurrent inhibition was disabled to isolate the quantization effect and assess the initial recruitment of MCs and GCs. MC → GC synapses were trained using two-shot learning for each of the eight presented analytes prior to assessment (see *Methods*), so that GC response profiles would statistically resemble those of a mature EPL network.

### Heterogeneous quantization regularizes spiking activity and mitigates information loss

Biological systems deploy diverse mechanisms to accommodate natural inputs with wide dynamic ranges. Among these strategies, heterogeneity (e.g., of connection topologies or synaptic weights) is prominently employed across sensory systems^19,27–29^. In the mammalian olfactory bulb, tens of principal neurons are associated with each column, inheriting afferent input from the same primary sensory receptor class. We tested whether implementing heterogeneity across these duplicated parallel pathways—multiple MCs per column (Fig. 1)—could help regularize layer utilization at the quantization step without resorting to foreknowledge of environmental statistics or a time-consuming hyperparameter optimization procedure. Specifically, we studied the effects of heterogeneity among within-column ET → MC synaptic weights and MC spike thresholds (see *Supplementary Data*); we here focus on the former as it better segregates this transformation from subsequent computations within EPL^18^.

To obtain a set of baseline measures, we first trained networks with *homogeneous* ET → MC weights (and uniform MC thresholds) on pure samples of simulated analytes from our two datasets. Network sizes varied, reflecting an MC column *duplication factor* of 4x, 8x, 16x, or 32 × in each of the eight columns, but ET → MC weights (gain levels) were all identical. The network was then tested with 10 possible weight values, drawn from a $\frac{1}{x}$ distribution (see *Supplementary Data*) and covering the input range $[.01, 1]$ across sensors. Training was two-shot (i.e., 16 samples in total, two from each analyte class), and eight-fold cross validation was used to obtain confidence intervals around the measures we report (see *Methods*).

**Fig. 3:**
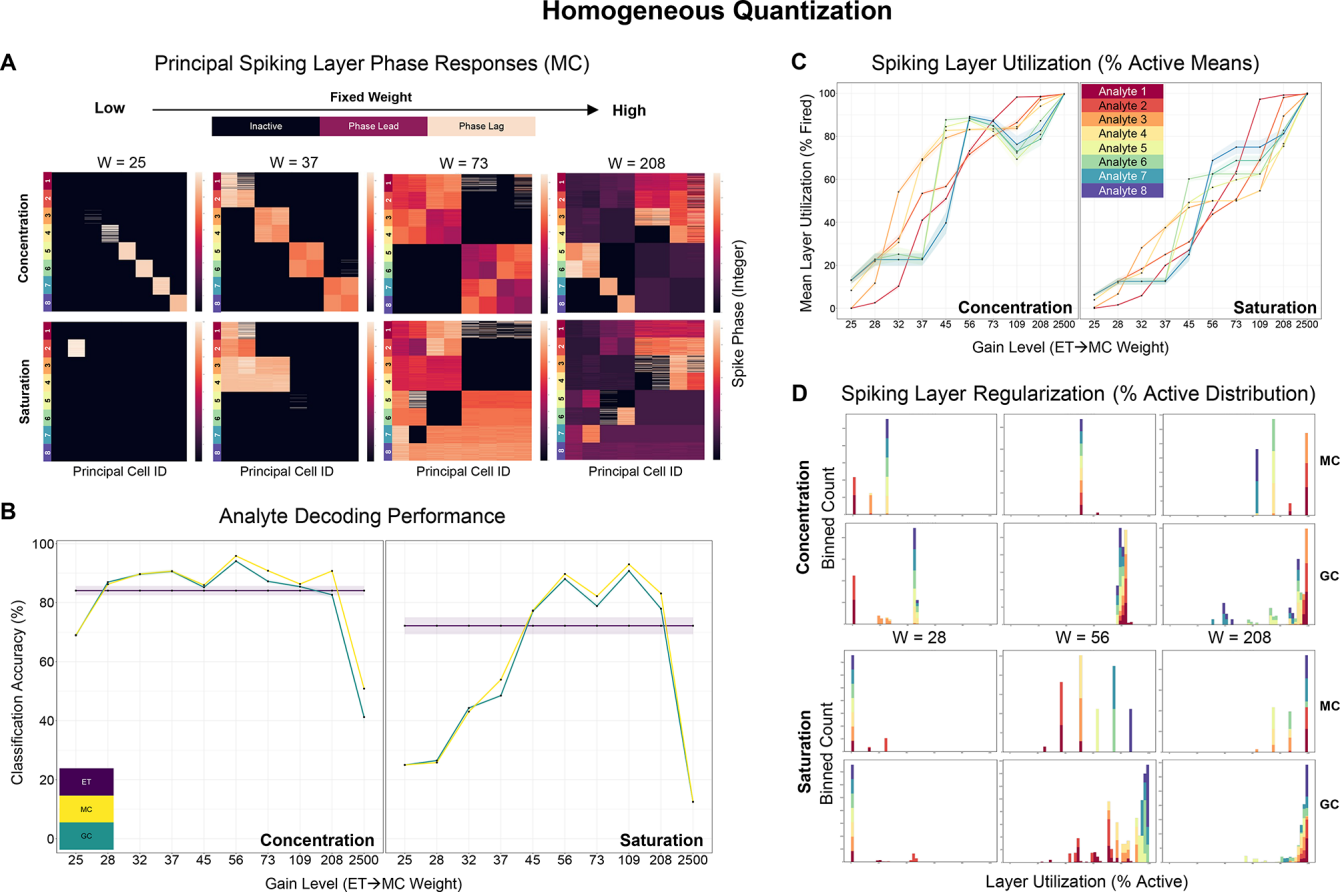
Outcome of homogeneous quantization. A fixed ET → MC weight value is adjusted as a control parameter on different simulation runs. Possible weights are distributed $\frac{1}{x}$ to emulate a set of equidistant MC thresholds covering the input range $[.01, 1]$ (see *Supplementary Data*). Results are aggregated over eight cross validation folds. (**A**) Principal layer (MC) spike phase responses to test samples from the two datasets. Spike phase responses range across $[1, 50]$ and inactive units are coded as zeros (black). Within each matrix, rows correspond to individual samples and are sorted by analyte class. Columns correspond to individual MCs. (**B**) Line plots depicting classification performance of hyperparameter optimized RBF SVMs trained separately on ET (purple), MC (yellow), or GC (teal) phase responses, illustrating the effects of increasing gain levels (ET → MC weight) on information representation in homogeneous networks of varying sizes (4 × to 32x; see *Methods*), in the presence of concentration variability and sensor saturation. ET responses function as a baseline, being identical to the normalized input test samples. (**C**) Spiking layer utilization, averaged across models and over the MC and GC layers. The curves depict the mean percentage of active units as gain increases; line colors correspond to analyte class labels. (**D**) Stacked histograms depicting MC and GC layer utilization (%) as gain increases, for the concentration (*top*) and saturation (*bottom*) datasets, at 32 × duplication. Across figures, ribbons represent the standard error of the mean over eight cross validation folds. Abscissa scale is from 0 to 100% in all panels; colors correspond to analyte class labels.

Figure 3A depicts MC spike phase responses across four of the ten ET → MC gain levels (*W*), illustrating that no single weight can generate a high-quality representation of all tested analytes. To quantify this, we measured the mean analyte classification accuracy for each dataset across a range of fixed ET → MC synaptic weights. We trained separate support vector machines (SVM) with a radial basis function (RBF) kernel (see *Methods*) on MC and GC phase response vectors as well as on ET activation levels (equal to the normalized analog input vectors, and serving as a baseline). In the concentration dataset, information was lost only at the lowest and highest weights, whereas in the saturation dataset, information was fully retained, on average, only within a narrower weight range (Fig. 3B).

Increasing ET → MC gain also broadened the activation of MCs (Fig. 3B). Examining the analyte-specific recruitment of MCs across these different weights revealed that, even at moderate weights, some analytes activated nearly the full complement of MCs (Fig. 2B, 2F)—an undesirable state even if reliable phase differences among activated MCs were maintained. Finally, and most critically, even within single gain levels (*W*) and despite GlomL data normalization, different analytes recruited widely different numbers of MCs and GCs from the same parameterized network (Fig. 3D). This high variance in neuronal recruitment disrupts the balance between excitation and inhibition required for robust neuromorphic network operation; if the network was well parameterized for some analytes, it necessarily would be poorly parameterized for others.

Overall, no optimal quantization parameters could be found that would consistently preserve similarity structure, retain analyte decodability, and ensure the stability of EPL network dynamics and plasticity across analytes and datasets.

**Fig. 4:**
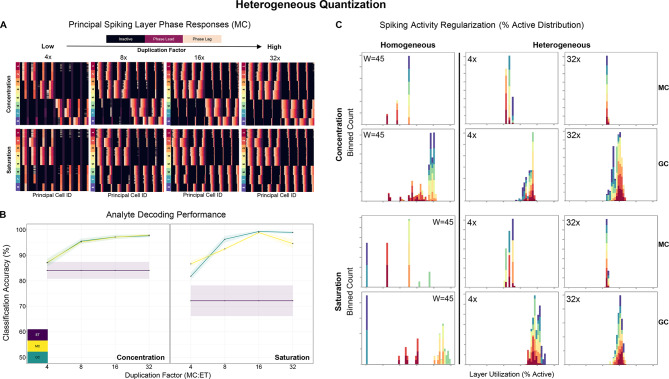
The effect of heterogeneous quantization on regularization and information at four duplication factors: 4x, 8x, 16x, 32x (corresponding to 32, 64, 128, and 256 MCs, respectively). The weights of synapses originating from the same ET cell are distributed $\frac{1}{x}$ to emulate equidistant MC thresholds covering the input range [.01, 1] within each MC column (see *Supplementary Data*). Results are aggregated over eight cross validation folds; ribbons represent the standard error. (**A**) MC spike phase response matrices at each duplication factor, separated by dataset. (**B**) Line plots depicting classification performance of hyperparameter optimized RBF SVMs trained separately on ET (purple), MC (yellow), or GC (teal) phase responses, demonstrating a marked increase in information across layers as the duplication factor increases. ET responses are equivalent to the normalized input test samples and serve as a baseline. (**C**) Stacked histograms depicting MC and GC layer utilization (%) for two heterogeneous conditions (4 × and 32x; *rightmost columns*), alongside their 32 × homogeneous counterparts (mid-level gain of *w* = 45 depicted, *leftmost column*). Heterogeneous weights yield consistent utilization of the MC and GC layers irrespective of analyte identity; the increasingly narrow distributions of neuron recruitment levels as weight heterogeneity increases illustrate the correspondingly improved statistical regularity of EPL sensory representations (cf. Figure 3D). Abscissa scale is from 0 to 100% in all panels; colors correspond to analyte class labels.

We sought a transformation in which a single parameterized network could encode this full diversity of analytes within a predictable and balanced regime. We therefore repeated the above simulation experiments with *heterogeneous* ET → MC weights, at MC duplication factors of 4x, 8x, 16x, and 32x. The weights were similarly $\frac{1}{x}$ distributed and covered the same input range, but this time the weight variance was deployed across multiple MCs within each column rather than across simulation runs. This yielded more balanced spike phase responses (in Fig. 4A, on the abscissa, MCs with a common ET afferent are adjacent and sorted by weight, low to high). To assess the performance of this strategy, we fit general linear models (GLM) to the dependent variables *information* and *regularization*, with *condition* (homogeneous or heterogeneous)*, duplication factor*, *layer* (MC or GC), and *dataset* as fixed factors, and *cross validation fold* as a random factor. Unless otherwise stated, general linear contrasts were Tukey-corrected within (but not across) nested levels of the relevant factors.

In the model fit to *classification accuracy*, we observed main effects for all factors $F([1, 3], 217) > 9.372$, $p < .0025$. In addition, except for *layer* by *dataset* and *condition* by *layer* by *dataset*, all two-way and higher order interactions were significant, $F([1, 3], 217) > 2.702$, $p < .0465$. Planned contrasts revealed that analyte classification accuracy was higher with heterogeneous than with homogeneous ET → MC weights when either MC responses ($t(217) > 21.773, p < .0001$) or GC responses ($t(217) > 22.268, p < .0001$) were used as input vectors to the classifier (Fig. 4B). In the *regularization* model, all main effects were significant, $F([1, 3], 217) > 4.266, p < .006$. Two-way interactions of *condition* by *layer* and *condition* by *dataset* also were observed, $F(1, 217) > 9.739, p < .002$. Specifically, the number of active MCs was more consistent across diverse test samples in the heterogeneous than in the homogeneous models; across all duplication levels, the standard deviation of MC and GC utilization was lower (better) when variable ET → MC weights were used ($t(217) > 19.026$, $p < .0001$). These findings indicate that, all else being equal (including network size), a data-blind $\frac{1}{x}$ distribution of ET → MC quantization weights within each column regularizes neuronal activation and enhances information retention in downstream spiking layers (Figs. 4C, S2, S3). That is, heterogeneity in this quantization step enables the construction of a statistically well-behaved sensory representation within the EPL layer of the OB network.

### Adaptive quantization jointly optimizes resolution and activity regularization

A $\frac{1}{x}$ distribution of ET → MC weights within each column (or a uniform distribution of MC thresholds; see *Supplementary Data*) provides data-blind optimal regularization of unregulated sensory inputs, thereby presenting statistically well-behaved inputs to the spiking network of the EPL (Fig. 1); higher duplication factors generate correspondingly more reliable distributions (Figs. 4C, S2). This flat prior is generally responsive to all possible sensory input profiles. However, to the extent that the statistics of a test environment are predictable, a data-aware modification of this approach can maintain or improve performance while expending fewer resources (i.e., achieving a given degree of information retention with a lower duplication factor). If the full potential activation range of a given sensor (here, $[0, 1]$) is not predicted to be used within a test environment, mapping only the range of expected activation levels will increase resolution at the cost of returning a low-quality representation should this range be exceeded. Second, the likelihood distribution of particular activation levels of each sensor within a given environment can be learned, and the distribution of the quantization weights mapped accordingly, assigning higher resolution to the most probable or most task-important ranges of sensor activation.

This is a deeply biological design principle. Brains, over behavioral, developmental and evolutionary timescales, adapt to the statistics of their input and exploit regularities to optimize processing. This manifests in functional architectures that vary widely across sensory systems. Moreover, biological circuits conserve resources by allocating them selectively, in both the structural (e.g., a greater density of cone photoreceptors in the fovea than at the periphery^30,31^) and functional (e.g., saccading behavior^32^) senses. In many artificial systems, it is desirable to allocate bandwidth on demand; here, we leverage this design principle to minimize resource allocation while jointly optimizing information retention and activity regularization.

In this final set of simulation runs, we introduced an adaptive pre-training calibration step, utilizing the same small set used for two-shot training. ET → MC weights, which were uniformly spaced ($\frac{1}{x}$) in the standard heterogeneous condition, now were allowed to vary in range and density (independently across MC columns) to match the distribution of the calibration observations; in other words, we used the calibration set to construct a non-flat prior on the input distribution. We tested two variants of the calibration procedure: in the *scaled* condition, weights covered the range between the maximal and minimal observation recorded by each sensor in a $\frac{1}{x}$ distribution; in the fully *adaptive* condition, weights were piecewise interpolated to match the calibration set distribution, and the number of segments (dictating the smoothness of the interpolation) was identical to the duplication factor (see *Methods*). We hypothesized that adaptation would enhance the system’s capacity for discrimination at lower duplication factors while sacrificing some degree of regularization (i.e., the standard deviation of layer utilization would be somewhere in between the homogeneous baseline and the generic heterogeneous condition).

As before, we fit GLMs to our *information* and *regularization* measures, with *condition* (this time with four levels: homogeneous, heterogeneous uniform, scaled, and adaptive), *duplication*, *layer* (MC or GC), and *dataset* as fixed factors, as well as *cross validation fold* as a random factor.

**Fig. 5:**
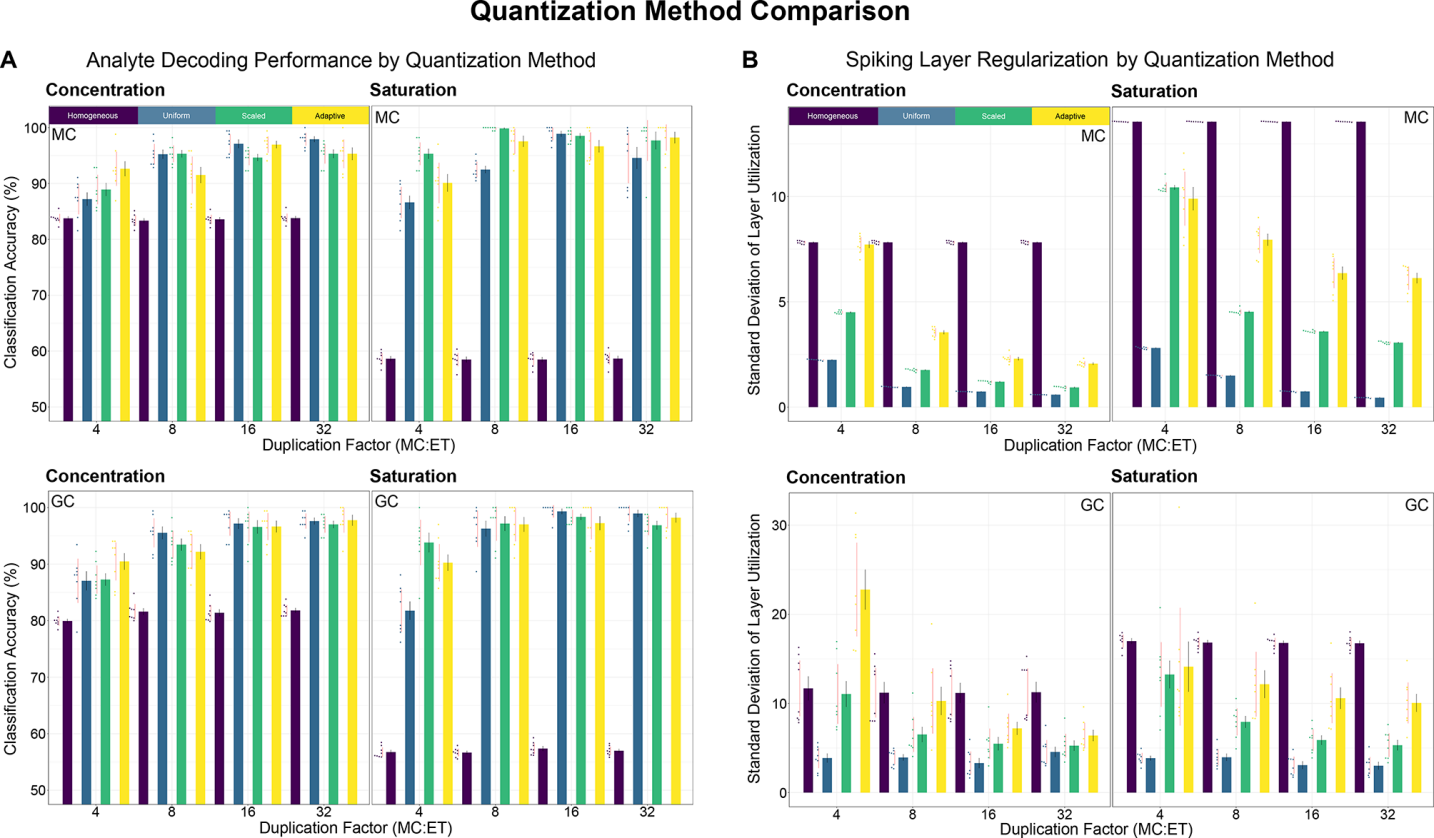
Calibration optimizes resource utilization and trades off regularization for enhanced resolution in ranges where observations are expected to be found. Heterogeneous quantization weights encode a non-uniform prior on the input distribution. Weights are interpolated along segments whose boundaries are cluster centers on the line (Jenks natural breaks, given the calibration observations for each sensor^33^). The number of segments is controlled by a smoothing factor. In the scaled condition, a single segment is used, bounded individually for each sensor by the minimal and maximal values recorded. In the adaptive condition, multiple segments are used, yielding a better fit to the calibration data. (**A**) Classification performance of cross validated and hyperparameter optimized RBF SVMs, trained separately on MC and GC phase responses from individual models with different duplication factors. (**B**) Regularization of layer utilization (standard deviation of percentages of active MC and GC neurons), illustrating the regularization-information trade off as a function of the MC duplication factor. In all panels, bars depict mean classification accuracies within each layer, condition, and level of duplication factor. Black ticks represent the standard error of the mean. Red vertical lines correspond to 95% confidence intervals around each mean. Scattered points are individual accuracy observations, each from one of eight cross validation folds.

In the model fit to classification accuracy (Fig. 5A), we observed main effects for every factor, $F([1, 3], 441) > 5.805$, $p < .0164$. Except for the *layer* X *dataset* interaction and all three-way interactions involving *layer*, every interaction effect was significant, $F([1, 3, 9], 441) > 2.719$, $p < .0273$. Across duplication factors and layers, all heterogeneous condition variants yielded higher classification accuracy compared to their homogeneous counterparts, $t(441) > 18.254$, $p < .0001$. At the lowest duplication level, 4x, the scaled and adaptive variants yielded boosted accuracy relative to the uniform heterogeneous condition, $t(441) > 5.188$, $p < .0001$, illustrating the compensatory effect of data-aware calibration. At 8 × duplication, only MCs showed an advantage for the scaled compared to the uniform heterogeneous condition, $t(441) = 4.32$, $p = .0001$. By 16 × and 32x, all heterogeneous networks had reached ceiling performance, yielding no differences among the uniform, scaled, and adaptive variants, $t(441) < 1.643$, $p > .3556$. In the heterogeneous conditions (but, critically, not in the homogeneous baselines; $p > .6941$), increasing duplication from 4 × to 32 × provided a considerable boost to information retention; for both MCs and GCs, classification accuracy increased monotonically with the duplication factor. The largest difference was observed between 4 × and 8 × across conditions, $t(441) > 3.675$, $p < .0007$, followed by 8 × and 16 × in all conditions except *scaled*, $t(441) > 2.508$, $p < .0347$. By the final step, 16 × to 32x, an asymptote had been reached across conditions and layers, resulting in nonsignificant differences, $t(441) < 2.032$, $p > .1121$.

When measuring the regularization of layer utilization (Fig. 5B), we found main effects of all factors, $F([1, 3], 441) > 91.858$, $p < .0001$. With the exception of *duplication* X *dataset*, $F(9, 441) = 2.041$, $p = .1074$, all interactions were significant, $F([\text{1,3},9], 441) > 2.721$, $p < .0059$. General linear contrasts revealed that, across spiking layers, utilization regularization was worse (more variability) in the homogeneous condition relative to the three heterogeneous conditions, $t(441) > 2.625$, $p < .0442$, with one exception; for GCs, at 4 × duplication, the adaptive condition was worse than the baseline, $t(441) = 5.735$, $p < .0001$. For GCs, utilization variability was higher in the scaled and adaptive conditions relative to the uniform heterogeneous condition at duplication levels 4x, 8x, and 16x, $t(441) > 3.77$, $p < .0001$. By 32x, utilization was similarly regularized in the uniform and scaled variants, $t(441) = 2.101$, $p = .1544$, and both showed an advantage relative to the fully adaptive variant, $t(441) > 4.104$, $p < .0003$. For MCs, worse regularization was observed in the adaptive compared to the uniform variant across duplication factors, $t(441) > 4.995$, $p < .0001$. That was also true for the scaled relative to the uniform condition at 4 × and 8x, $t(441) > 2.677$, $p < .0385$, and for the adaptive relative to the scaled condition at all duplication levels, $t(441) > 2.697$, $p < .0364$, except 4x, $t(441) = 1.872$, $p = .2416$, consistent with the existence of a regularization-resolution tradeoff. The above outcomes demonstrate that the benefits of heterogeneous quantization at modest resource multipliers can be enhanced by adopting a data-aware quantization strategy. With lower duplication, modifying the range and distribution of ET → MC weights on a sensor-by-sensor basis, based on calibration observations, significantly boosted information retention with a small negative impact on spiking activity regularization. This property can be useful with denser sensor arrays, where using higher duplication factors can become resource intensive.

## Discussion

The goal of odor source separation and identification from real-world data presents a challenging problem. Whereas some simple tasks can be performed by analyte-specific electrochemical sensors (e.g., CO_2_ detectors) that exhibit negligible sensitivity to background sources^34^, most chemosensors are responsive to broad ranges of chemical analytes with related properties. Accordingly, biological and most artificial chemosensory systems pursue the richer strategy of deploying arrays of partially selective sensors; these chemosensor arrays achieve response specificity in combination and enable the assessment of similarity between sources. Because both individual odors of potential interest and multisource odor scenes constitute linear combinations of analytes present at different concentrations, the mixing of these analytes can exert nonlinear and even nonmonotonic effects on cross-responsive chemosensors^6^, which effectively occlude diagnostic activity patterns across the array.

Neuromorphic algorithms, inspired by specific computational strategies of the mammalian olfactory system, have been trained to rapidly learn and reconstruct arbitrary odor source signatures from chemosensor array data in the presence of some forms of background interference^18^. However, such networks perform best when tuned to the statistics of well-behaved inputs, normalized and predictable in their activity distributions. Deployment of chemosensor arrays in the wild exposes these networks to additional, powerfully disruptive effects, including (1) high variance in analyte concentrations, which is nonlinear and dwarfs the quality-based signal variance upon which source identification depends, and (2) odor environments that range from sparse to richly complex, yielding varied distributions of activity levels across different sensors in the array even after normalization. Regularizing spiking activity distributions is a prerequisite for more sophisticated sensory constructions, such as hierarchical category learning^35^.

To address these challenges, we expanded and unified our previous neuromorphic models of the olfactory bulb glomerular layer^1,26,36^ and of the EPL^18^, the latter leveraging gamma-like inhibitory oscillations^37–40^ to induce a spike-phase code and partition subsequent stimulus processing into cycles. Like the biological system that inspired it, the present network routes sensor outputs to ET cells^41^ within a glomerular layer circuit that normalizes activity by lateral shunting inhibition (division)^23^. Here, we investigated the computational properties and effects of the quantization step, where the normalized analog representations of the ET cell layer are transformed into the spike phase vectors of the MC layer. The choice of analog-to-spiking synaptic weights is critical, as it constrains the dynamic range and resolution of downstream representations. Our goal was to jointly optimize information (discriminability), spiking activity regularization, and resource utilization.

We deployed a spectrum of *heterogeneous quantization* strategies, all involving the use of variable ET → MC weights; this mechanism amounts to gain diversification among parallel outputs (as in spare receptor theory^19^), enabling downstream layers to represent signals spanning a wide dynamic range while minimizing information loss and avoiding a combinatorial explosion of resources. The quantization weights can be conceived of as a prior on the input distribution. In the *uniform* variant of the heterogeneous condition, the weights were $\frac{1}{x}$ distributed (equivalent to linearly spaced MC thresholds; see *Supplementary Data*) and covered the full range of normalized inputs within each MC column, corresponding to a flat prior on each sensor’s response profile. This yielded optimal spiking activity regularization across test samples and a marked improvement in the information decodable from MC and GC spike phase responses compared to the homogeneous baseline.

While the generality of the flat prior achieves the primary goal of enabling a single parameterized network to be well adapted despite the unpredictability of open-set sensor deployment in the wild, it also is true that adapting to the statistics of the chemosensory environment can yield superior performance at lower cost, particularly with smaller duplication factors. To characterize this option, we calibrated the distribution of the ET → MC quantization weights to the environment; specifically, the weights were allocated more densely to ranges in which data were more likely to be found, based on the individual sensor responses observed in a pre-training calibration step. For richly structured datasets, such as those used in the present study, this approach can improve fine discrimination capacity without excessive resource scaling and with a relatively small impact to regularization metrics. This tuning method amounts to piecewise interpolation and enables the user, by adjusting the number of segments (i.e., a smoothing factor), to regulate bias and variance in the model.

Learning sensory representations online, under natural conditions, involves a quantization problem whose outcomes are sensitive to gain modulation. This is especially true in the presence of large sensor arrays, multiscale structure, and weakly diagnostic features. The choice of quantization weights, implicit in many models, determines what orders of magnitude can be represented in the spiking activity of downstream layers. Since the relevant scales are typically not known a priori, the deployment of heterogeneous parallel paths—whether data-blind or data-aware—can provide principled solutions with quantifiable bounds. Heterogeneous quantization is a viable alternative to intensive hyperparameter grid searches and is particularly suited to intelligent edge devices.

## Methods

### Synthetic data generation

Artificial input vectors were generated that emulate the dynamic range and saturation problems commonly observed in artificial chemical sensors deployed in the wild^42,43^ and in the statistics of olfactory sensory neuron (OSN) responses.

*Affinity Matrix*. We designed an affinity matrix ${G}_{8 x 8}$ (sensor by source) defining the similarity structure of an artificial odorant space. Values were picked so as to induce a balanced, three-level category hierarchy among eight simulated analyte classes. Receptive fields were distributed symmetrically, such that each analyte had one sensor highly sensitive to it, while others (primarily responsive to other sources) were weakly to moderately sensitive to it (see *Supplementary Methods*). The sparsity of the affinity matrix was defined as the Hoyer normalized sparsity measure, $\frac{1}{\sqrt{N}-1}(\sqrt{N}-\frac{{l}_{1}}{{l}_{2}})$^44,45^, applied to each sensor’s affinity vector. The mean sparsity of the affinity matrix $G$ was fine tuned by iterative element-wise exponentiation followed by row-wise ${l}_{1}$ normalization, ${{{\forall }_{0 \le i < n} G}_{i} \leftarrow }\frac{{{G}_{i}}^{\alpha }}{|| {{G}_{i}}^{\alpha }|{|}_{1}}$, until an overall Hoyer measure of 0.4 was arrived at, emulating moderately selective artificial sensors. This transformation alters the original similarities but preserves their rank order; it enables the design of hierarchical discrimination problems with a high degree of control over synthetic sensor properties, receptive field distributions, and source mixing proportions.

*Concentration Dataset*. To generate a dataset containing samples with large systematic differences in dynamic range, we multiplied the affinity matrix $G$ by a set of eight-element vectors ${x}_{k}$ corresponding to pure artificial odorant samples whose concentrations were exponentially distributed with analyte-characteristic means. To render the classification problem more difficult, we introduced additional variance among samples by injecting a small amount of Gaussian noise to the responses, centered at zero and varying in scale across sensors (for exact parameterization, see *Supplementary Methods*).

*Saturation Dataset*. The total output emitted by a biological OSN unit can be thought of as the sum of multiple sigmoid activation functions with a scaling parameter $s$ and a translation parameter $t$^19^. This can yield systematic sensor saturation effects under some conditions. We generated an additional dataset modeling this problem by applying a sigmoid transform to the concentration set samples (for exact parameterization, see *Supplementary Methods*). For both datasets, the noise standard deviation was selected so as to render the classification problem nontrivial but tractable. Other free parameters were chosen to isolate and exaggerate the geometric properties of interest.

### Network architecture

Network structure followed the circuit motifs of the mammalian olfactory bulb^21^, extending and modifying past iterations of our *Sapinet* model^1,18,36^. The present network combines a spiking representation learning module, corresponding to the bulbar *external plexiform layer* (EPL), with an analog signal conditioning module inspired by the *glomerular layer* (GlomL). Here, all weights and states were simulated with float32 precision on an Intel i9-14900 k CPU. However, both network components abide by neuromorphic design principles and can be deployed on neuromorphic platforms^18^.

#### Neurons and connection topology

All spiking units in the model were leaky integrate-and-fire (LIF) neurons, integrated using the fourth order Runge–Kutta method with an update time constant of $1 ms$. The evolution of the membrane potential was governed by the dynamical equation ${\tau }_{m}\frac{dv}{dt}=\frac{I}{{g}_{L}}-(v-E)$, where $v$ is the membrane potential, ${\tau }_{m}$ the membrane time constant, $I$ the input current, ${g}_{L}$ the leak conductance, and $E$ the resting potential. Membrane potential was reset to $E$ upon exceeding a voltage threshold ${v}_{th}$.

The signal conditioning portion of the network, tasked with normalization and quantization, consisted entirely of analog neurons (for an input current $I$, $\frac{dv}{dt}=I$). Olfactory sensory neurons (OSN) represented sensor responses and directly corresponded to the elements of the input vectors. 8 OSNs projected to 8 external tufted (ET) cells in a one-to-one pattern. PG cells, depicted in Fig. 1, were omitted from the present analysis for simplicity.

Mitral cells (MCs) constituted the principal excitatory spiking layer. They followed a columnar organization principle, where each MC within a column received its primary external input from one ET cell, corresponding to a unique artificial sensor (OSN). All MCs were identically parameterized, ${\tau }_{m}=4$, ${g}_{L}=1$, $E=-60$, ${v}_{th}=-55$ (fixed).

Depending on model parameterization, there could be several MCs per column, enabling gain diversification through weight or threshold heterogeneity. In this experiment, the ratio of MCs to ETs (*duplication factor*) could be fixed at 1, 4, 8, 16, or 32 (yielding 8, 32, 64, 128, or 256 MCs, respectively). In some cases, ET → MC synapses within the same column varied in weight values, spanning different ranges in different experimental conditions (for details, see *Experimental Design*).

Granule cells (GCs) comprised a second spiking layer. Across conditions, there were four times as many GCs as there were MCs, resulting in a shallow and wide architecture conducive to the learning of basic chemosensory features. All GCs were identically parameterized, ${\tau }_{m}=4$, ${g}_{L}=1$, $E=-60$, ${v}_{th}=-50$ (fixed). GCs received random input projections from the MC layer, with MC → GC connection probability kept proportional to the ratio of MCs to ETs, $p = \frac{1}{MC/ET}$.

#### Oscillations and temporal dynamics

MCs received, at all times, additive sinusoidal input in the range $[-40, 0]$ with a period of $50 ms$ (simulation steps), segmenting the activity and allowing for iterative convergence of spike-phase representations to an attractor^18^. Accordingly, all spiking unit voltages and refractory periods were reset every $50 ms$. By enforcing a refractory period, all spiking units were forced to fire only once within each cycle of the background oscillation.

This mechanism follows the example of gamma oscillations in the olfactory bulb^40^, by which periodic inhibition synchronizes spiking activity across columns^37–39^. In this mechanism, alternating spiking and quiescent periods enable a within-cycle spike precedence code to form, in which earlier spike phases correspond to stronger inputs.

#### Synapse models and learning rules

In the GlomL, connections between OSNs and their respective ETs were simple relays ($w=1$). ET cells were fully laterally connected with shunting synapses (including self-connections); this divisive inhibition implemented ${l}_{1}$ normalization, ensuring $ET(x) = \frac{x}{||x|{|}_{1}}$ for any input vector $x$.

The ET → MC quantization synapse weight distributions varied across experimental conditions. In the uniform heterogeneous condition, $\frac{1}{x}$ distributed weights were chosen to cover the normalized input range $[.01, 1]$ (that is, to allow at least one MC to fire for an input at the network’s detection limit, here set to 0.01, subject to LIF parameterization). In others, weights could cover more limited input ranges or vary in density (see *Experimental Design*).

MC → GC synapses were additive. Their weights were initialized to $w=25$ and updated during training in accordance with a standard Hebbian STDP rule, $\Delta {w}_{ij}= {\alpha }_{+} \bullet {e}^{{\frac{{t}_{j}-{t}_{i}}{{\alpha }_{+}}}}$ for potentiation, $\Delta {w}_{ij}= {-\alpha }_{-} \bullet {e}^{{\frac{{t}_{i}-{t}_{j}}{{\alpha }_{-}}}}$ for depression, where ${\tau }_{+}, {\tau }_{-}=4$, ${\alpha }_{+}= .3125$, ${\alpha }_{-}= 1.25$^18,25^. Updates were performed on every cycle of the background oscillation, as spiking neurons were guaranteed to fire at most once. Learned weights were clamped to the range $[0, 30]$, and a half-cycle synaptic delay ($25 ms$) ensured GCs could only fire when MCs were quiescent.

### Experimental design

We tested the effects of the quantization transformation (from analog ET responses to MC spike phase representations) on the following dependent variables: (1) variability in spiking layer utilization (*regularization*); and (2) the quality of the learned representations (*information*). We introduced varying levels of heterogeneous ET → MC weight duplication, comparing layer utilization and information measures to a homogeneous control condition in which network topology was matched but all ET → MC connections had identical weights. We also altered the distribution of ET → MC quantization weights based on the statistics of a calibration set, with the aim of demonstrating that utilization regularization can be traded off for enhanced resolution in a target range. These objectives yielded four experimental conditions, contingent on two key independent variables: (1) ET → MC weight distribution; and (2) duplication factor (MC:ET ratio; 4, 8, 16, or 32).

In the *homogeneous* condition, the number of MCs was identical to the number of ETs (8); one of 10 weights was picked out of a $\frac{1}{x}$ distribution covering the input range $[.01, 1]$ (with respect to the chosen LIF parameters for MCs) and applied to every ET → MC synapse. The MC duplication factor varied among the levels 4x, 8x, 16x, and 32x, and samples could be drawn from the concentration or the saturation datasets. Thus, this design yielded a total of 80 homogeneous scenarios, each corresponding to an independent simulation run.

In the three *heterogeneous* conditions, duplicated MCs were arranged in columns defined by having a common ET afferent; ET → MC weights within each column were varied. Each heterogeneous condition yielded 8 independent simulation runs (4 duplication levels, 2 datasets), for a total of 24 runs. In the *uniform* variant, ET → MC weights covered the input range $[.01, 1]$. In the *scaled* variant, they covered the range $[min({x}_{i}), max({x}_{i})]$ across calibration set observations $x$. The *adaptive* variant was similar to the *scaled* one, except the densities of the ET → MC weights were selected to approximate the exact distribution of calibration observations for each sensor, with a certain degree of smoothing. Specifically, within each duplication factor level $n$, each sensor’s range was partitioned into $n$ segments. Optimal partition points were determined using one-dimensional clustering (Jenks natural breaks optimization)^33^; the same number of weights was assigned to each segment despite potential differences in width, yielding a variable density distribution reflecting the statistics of the held out set used for training and calibration.

To quantify spiking layer activity *regularization*, we computed the standard deviation of the percentage of units that spiked in response to each test sample, separately for the MC and GC layers within each network model. Minimizing the variance in spiking layer utilization across input samples stabilizes representations and plasticity mechanisms, maintaining EPL network activity in the sensitive, well-tuned midrange and also ensuring that concentration differences are not mistaken for analyte properties and exploited for discrimination. To quantify the *information* encoded in the learned EPL representations, we fed MC and GC responses to a cross validated, hyperparameter optimized support vector machine (SVM) classifier with a radial basis function (RBF) kernel (for details, see *Procedure*). Classification accuracy was used as a measure of whether the transformation (i.e., analog input vectors to MC and GC spike phases) resulted in more (or less) easily separable representations, using the ET responses as a baseline (as those were equivalent to the normalized analog input vectors).

### Procedure

We performed a total of 104 simulation runs spanning four quantization conditions (see *Experimental Design*). Independent simulations were performed with a consistent random seed across the relevant libraries (*random*, *numpy*, and *pytorch*), ensuring reproducibility of the processing steps described below.

*Sampling*. At the beginning of each simulation run, 128 vectors were sampled from either the *concentration* or *saturation* dataset; samples were identical across independent instances of the pipeline. Within each instance, eight-fold cross validation was performed; an initial shuffling of the sample indices was followed by their partitioning into non-overlapping splits, each consisting of 16 training samples (two per analyte class, consistent with two-shot learning) and 112 test samples (14 per analyte class).

*Calibration*. In the *scaled* and *adaptive* heterogeneous quantization conditions, we introduced a pre-training calibration procedure. The network was exposed to the 14 training samples with synaptic learning turned off, and the range and density of ET → MC weights within each organizational column were updated (see *Experimental Design*).

*Model Training and Testing*. During training, the model was continuously exposed to each sample for 250 steps (corresponding to five oscillatory cycles), followed by a 50 step rinse cycle in which the network received an all-zeros vector as its input. MC → GC synaptic weights were updated at the end of every cycle in accordance with a Hebbian STDP rule (for parameterization, see *Network Architecture*). The same exposure and rinse durations were used in the test trials, with learning turned off. Response vectors (ET, MC, and GC) from the final (fifth) exposure cycle for each sample were logged and used in subsequent statistical and classification analyses.

*Classification*. To gauge the quality of the mapping learned by our EPL model, ET, MC, and GC spike-phase response vectors (*processed responses*) were fed, respectively, to three independent RBF kernel support vector machine (SVM) classifiers (using *scikit-learn*^46^). The following procedure was performed independently within each of the eight model-level cross validation folds (train-test splits): first, hyperparameter optimization was performed ($1{0}^{-7}<C<1{0}^{9}$, $1{0}^{-8} <\gamma <1{0}^{10}$, with logarithmically spaced intervals) using half (56) of the processed responses; then, SVMs were trained and tested on the remaining 56 samples. Stratified fourfold cross validation was used in both steps, with the smaller segment used as the internal training set (consistent with few-shot learning). Classification predictions were aggregated across these folds to yield confusion matrices containing prediction outcomes for 21 samples per analyte class (168 in total). Classification accuracy scores were calculated as the proportion of correct predictions out of the total number of processed responses in the test set. Thus, within each independent simulation run (corresponding to a single experimental condition) and layer (ET, MC, GC), 8 classification scores were obtained and subsequently used to compute 95% confidence intervals around the score mean.

## Software

Simulations were performed in Python 3.11 with *pytorch* 2.1.2 and *sapicore* 0.3.3. Sapicore is an open-source spiking neural network modeling framework developed by the authors to streamline the design and testing of neuromorphic algorithms on CPU/GPU. Sampling and classification were performed using *scikit-learn* 1.3.2. Statistical analyses were performed in R 4.1.2 using the packages *lme4*, *emmeans*, and *multcomp*. R plots were generated with *ggplot2* and *ggbeeswarm*.

## Supplementary Information

Supplementary Information.

## Data Availability

Source code for *Sapicore* is freely available on GitHub (https://github.com/cplab/sapicore). Our synthetic data, primary results, SVG figure elements, and statistics can be reproduced by running two master scripts (Python and R) provided in a separate repository accompanying this manuscript (https://github.com/cplab/sapinet_regularization); an environment file is provided for replication purposes, and the synthetic datasets are available in tabular form.
