# Supplementary material for: Heterogeneous quantization regularizes spiking neural network activity
Source: Sci Rep. 2025 Apr 23;15:14045. doi: 10.1038/s41598-025-96223-z (PMC12019593; doi:10.1038/s41598-025-96223-z)
Supplement: Supplementary file 1 — Supplementary Information. [file 41598_2025_96223_MOESM1_ESM.pdf]

Supplementary Methods

Data Synthesis

Affinities. To produce balanced correlated affinities inducing a nested similarity structure with three levels, we started with a matrix whose rows were identical to $G_{1,:}$ (below), then recursively swapped adjacent submatrices of decreasing sizes (powers of 2). The process yielded the following symmetric affinity matrix, used to generate both the *concentration* and *saturation* datasets:

$$G = \begin{pmatrix} 1 & .95 & .8 & .75 & .4 & .35 & .2 & .15 \\ .95 & 1 & .75 & .8 & .35 & .4 & .15 & .2 \\ .8 & .75 & 1 & .95 & .2 & .15 & .4 & .35 \\ .75 & .8 & .95 & 1 & .15 & .2 & .35 & .4 \\ .4 & .35 & .2 & .15 & 1 & .95 & .8 & .75 \\ .35 & .4 & .15 & .2 & .95 & 1 & .75 & .8 \\ .2 & .15 & .4 & .35 & .8 & .75 & 1 & .95 \\ .15 & .2 & .35 & .4 & .75 & .8 & .95 & 1 \end{pmatrix}$$

Sampling. Sensor response vectors sampled from the *concentration* dataset can be written as $OSN(x) = Gx + \varepsilon$, where ε is a Gaussian noise term $\sim N(0, \sigma_i^2)$ and x is a column vector with one nonzero element $x_i \sim exp(\lambda) + c$. The parameters σ, λ, and c were varied across, but not within, analyte classes i as follows: $c = (1,2,4,8,16,32,64,128)$, $\lambda = (10,10,1,1,1,1,1,1)$, $\sigma = (.01,.01,.01,.01,.01,.02,.05,.1)$, yielding the samples depicted in Fig. 2D. In the *saturation* dataset, the total output emitted by the sensors was $OSN(x) = \frac{1}{1+e^{-8(Gx-.5)}} + \varepsilon$, with the noise component parameterized $\varepsilon \sim N(0, .02)$ across analyte classes; other parameters were identical to the *concentration* set, resulting in the samples displayed in Fig. 2H. The resulting classification problem was nontrivial due to higher levels of either variance or additive noise in some analyte classes, but still retained a well-defined hierarchical similarity structure.

This parameterization yielded the following unsorted raw and normalized responses:

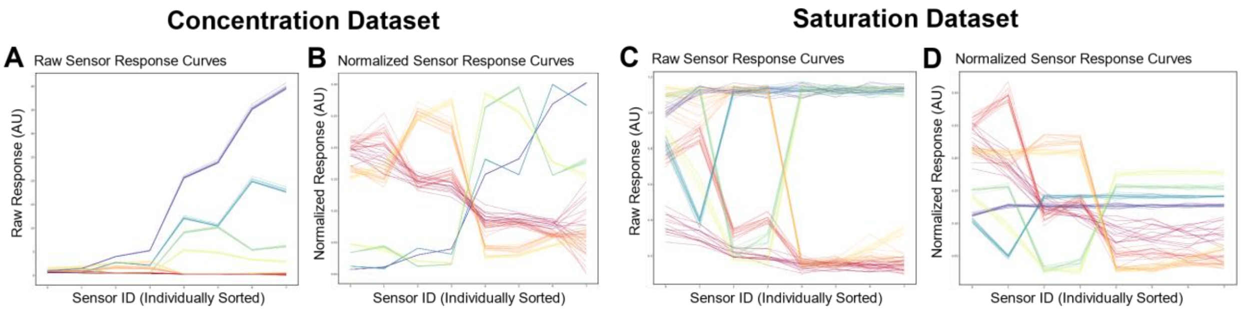

Supplementary Figure S1. Unsorted raw and normalized sensor responses to test samples from two synthetically generated datasets, accumulated over eight cross validation folds (train-test splits) and color coded by analyte class. (**A**) Dataset with balanced sensor affinities and systematic differences in synthetic analyte concentration. (**B**) A sigmoid transformed variant of the first dataset, with imbalanced response magnitudes and systematic differences in sensor saturation across analytes. (**C**,**D**) As A,B, but depicting data from the saturation dataset.

Supplementary Data

Statistical Analyses

The line plots below are an alternative presentation of the data in Fig. 5:

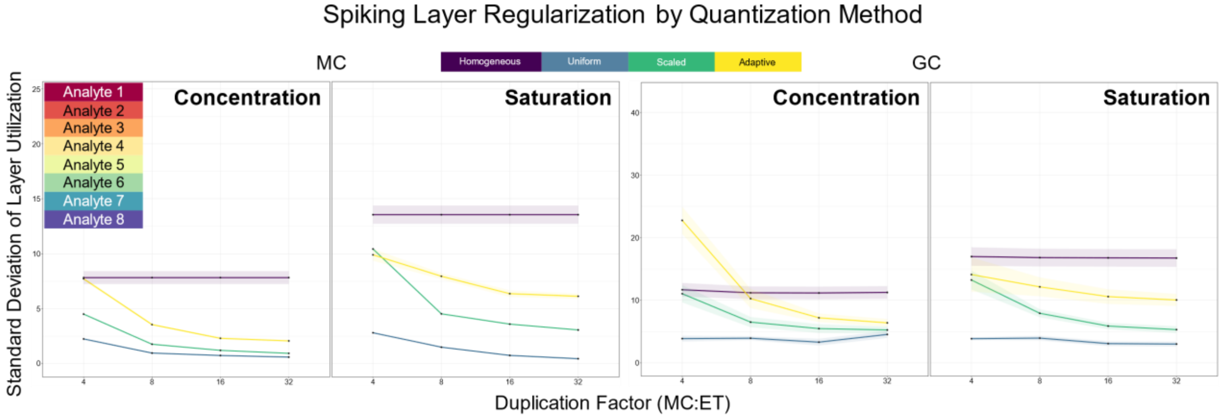

Supplementary Figure S2. Line plots depicting utilization regularization, defined as the standard deviation of the percentage of MCs (left) or GCs (right) that spiked in response to the presentation of analytes from the *concentration* or *saturation* datasets, as a function of the MC duplication factor (lower is better). Ribbons represent the standard error of the mean over eight cross validation folds. Colors denote the weight distribution strategy (see Fig. 5 for details).

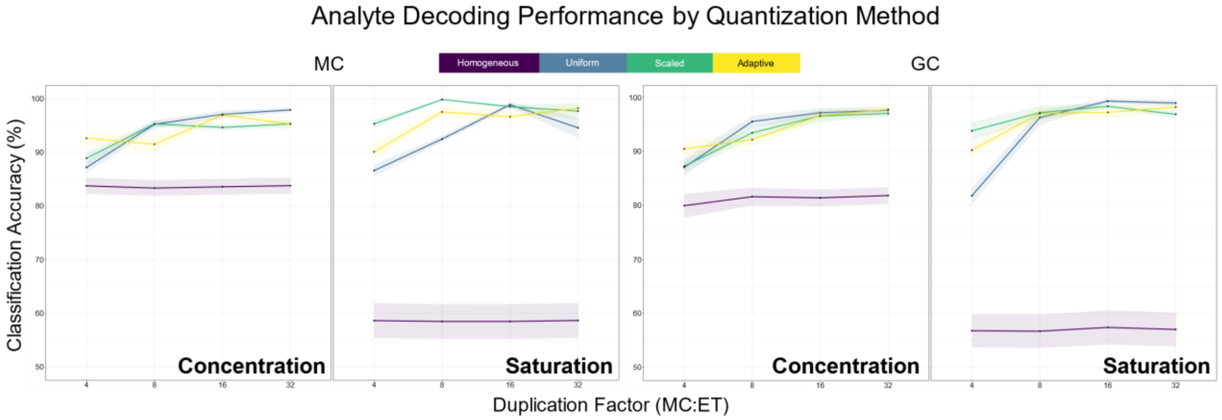

Supplementary Figure S3. Line plots depicting analyte classification accuracy based on MC (left) and GC (right) spike phase responses to the presentation of analytes from the *concentration* or *saturation* datasets, as a function of the MC duplication factor. Ribbons represent the standard error of the mean over eight cross validation folds. Colors denote the weight distribution strategy (see Fig. 5 for details).

Analysis of the Regularizing Effects of Gain Diversification

In the present neuromorphic model, non-spiking external tufted (ET) cells, of which there is one per column, deliver excitation onto spiking mitral cells (MC; Fig. 1). This is the quantization step, at which analog sensory inputs are transduced into spike timing-based representations. Each MC receives excitatory input from exactly one ET cell and is activated (spikes) if and only if this input exceeds its intrinsic spike threshold. In contrast, ET cells excite all of the MCs within their column, the number of which is determined by the duplication factor. Sensory inputs evoke activity in ET cells as follows:

$$A = [a_0 \;\; a_1 \;\; a_2 \ldots a_{n-1}]$$

where n is the total number of ET cells (corresponding to the number of columns) and a_i is the activity of the ith ET cell. As discussed in the Results, ET cell activity is normalized such that the total activation across the ET cell population sums to a constant c.

$$\sum_{i=0}^{n-1} a_i = c$$

For visualization purposes, we depict the activity of 8 ET cells (corresponding to 8 sensor-associated columns) as a line graph, sorted in descending order of the amplitude of their activation (Supplementary Fig. S4A; *black*). We will refer to this descending function as f. We can approximate the value of the area c under the function f as the Riemann sum of the areas of a number of discrete rectangles with finite widths (Supplementary Fig. S4A; *blue rectangles*):

$$Area(f) \ = \ \int_0^n f(x)\,dx \ \approx \ \sum_{i\prime=0}^{n\prime} f(x_{i\prime})\Delta x_{i\prime}$$

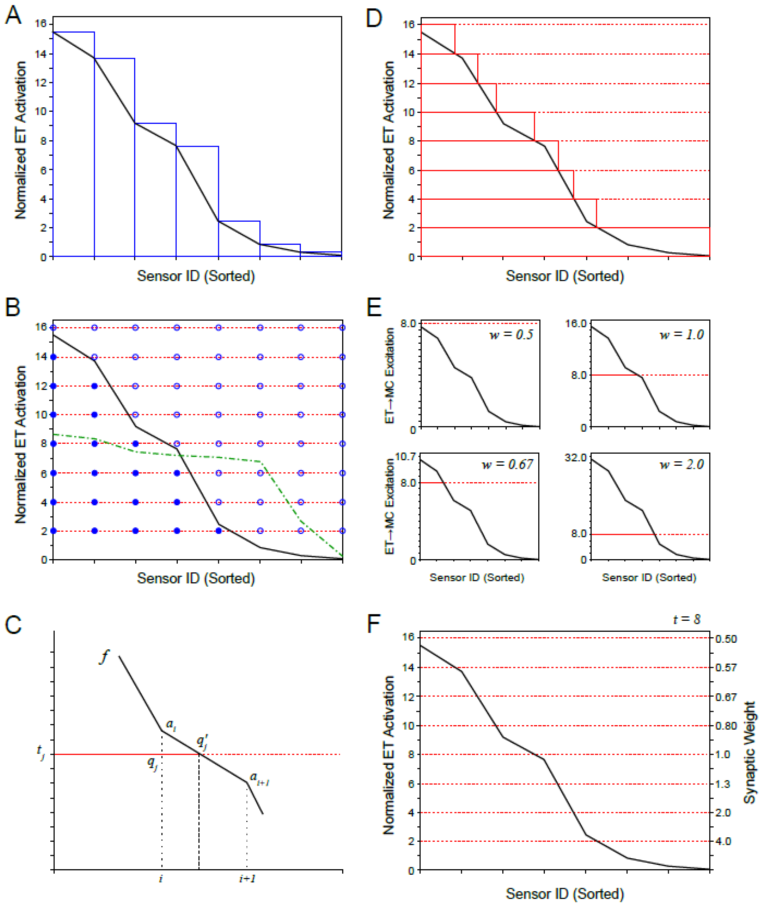

Supplementary Figure S4. (**A**) Normalized ET response profile of an analyte across 8 sensors (abscissa; cf. Fig. 2B, 2F), with Riemann subdivisions (*blue rectangles*). (**B**) The same response profile as in A, annotated with eight different, uniformly spaced, MC spike thresholds (*red dotted lines*; implies a duplication factor of 8). Each of the 8 sensor-associated ET cells activates one MC at each threshold, for a total of 64 MCs (*blue circles*). MCs below/to the left of the ET response profile (*black curve*) produce action potentials (*solid blue circles*). A normalized ET response profile from a different analyte (*green broken curve*) activates approximately the same number of MCs (*blue circles beneath green broken curve*), illustrating the regularization of total MC activity irrespective of input distribution. (**C**) Determination of the fraction of the MC population at a certain threshold that fires (*solid portion of red threshold line*), for a segment of the function f. (**D**) Same response profile as in A, annotated with eight subdivisions for calculation of the inverted Riemann sum (across thresholds rather than sensors). (**E**) Effect of heterogeneous ET→MC synaptic weights with a uniform MC threshold of 8. Greater weights generate larger numbers of activated MCs in the same way as lower MC thresholds do. (**F**) A $\frac{1}{x}$ distribution of synaptic weights yields the same outcome as a uniform distribution of MC thresholds.

Substituting the literal indices as $x_{i'} = i$, for which we know the value of $f(i) = a_i$, yields:

$$Area(f) \approx \sum_{i=0}^{n-1} f(i)\Delta x_i = \sum_{i=0}^{n-1} a_i \Delta x_i$$

Because the width of each rectangle is exactly one, and the sum of all ET cell activities is a constant:

$$Area(f) \approx \sum_{i=0}^{n-1} a_i$$

The approximation tends to the precise area under the curve as n increases.

Regularization of MC Spiking Activity via Heterogeneous MC Spike Thresholds

We first will demonstrate that heterogeneous MC spike *thresholds* will regularize the number of MCs activated by any sensory input distribution, given a constant area c under the function f of ET cell activation levels after normalization, and assuming that all ET→MC synaptic weights are identical and that the MC thresholds are uniformly distributed and span the entire codomain of f.

Assume that each ET cell projects to m MCs within its column (Fig. 1). Each of these MCs is assigned a different spike threshold, uniformly distributed between zero and some fixed upper bound u. The list of m thresholds will be the same in all columns. Hence, for n columns, there are n MCs exhibiting each threshold, and the total number of MCs in the network is nm. The assumption that the MC thresholds span the entire codomain of f means that $u \geq a_0$, though the general finding is robust to minor deviations from this assumption.

Consider ET_i, the ith ET cell with activity a_i. Let ET_i synapse onto $MC_{i,j}$ where i is the index of the presynaptic ET cell. Let $MC_{i,j}$ have threshold t_j. An $MC_{i,j}$ is considered to be active (spiking) if and only if $a_i \geq t_j$. A uniform distribution of eight MC thresholds is depicted in Supplementary Fig. S4B (*dotted red lines*), based on a duplication factor of 8.

We define the total number of MCs that are firing as Q. Since the the total number of MCs in the system is nm, the fraction of MCs that are firing is $\frac{Q}{nm}$. We want to demonstrate that this fraction is approximately constant for all normalized sensory inputs irrespective of their distribution (Fig. 2B, 2F). As MCs will spike if and only if their threshold is below the activity function f, it also is true, given that ET cell activation levels are sorted by amplitude, that MCs spike if and only if their thresholds are to the left of f (Supplementary Fig. S4B, *closed blue circles*). Heuristically, a different normalized input distribution, such as one drawn from the saturation dataset (Fig. 2F), still activates an approximately equal number of MCs (Supplementary Fig. S4B, *total count of the open and closed blue circles beneath the function g, depicted by broken green lines*).

To demonstrate this formally, Supplementary Fig. S4C depicts a section of function f. We define q_j' as the X coordinate of the point where f intersects the MC threshold t_j. Because only MCs with thresholds to the left of f will spike, $MC_{1,j}$, $MC_{2,j}$, ... $MC_{i,j}$ will spike and $MC_{i+1,j}$, ... $MC_{n,j}$ will not. That is, i out of n MCs at threshold t_j will fire. We therefore can define q_j as the (integer) number of spiking MCs at a given threshold t_j. In the above case, $q_j = i$. Because $|q_j' - q_j| < 1$, the value of q_j is always a strong (close) approximation of the value of q_j'. For large values of n, the fraction $\frac{q_j}{n} \approx \frac{q_j'}{n}$ represents the fraction of MCs that are spiking. That is, approximately q'_j MCs at each threshold t_j spike.

For a function like f that is non-increasing, with domain $[0, n]$ and codomain $[0, a_0]$, the Riemann sum can be used on either axis to estimate the area under the curve. That is, the regular Riemann sum and the inverted Riemann sum approximate the same area:

$$Area(f) = \int_0^n f(x)dx \approx \sum_{i'=0}^{n'} f(x_{i'})\Delta x_{i'} \approx \sum_{j=0}^{m'} f^{-1}(y_j)\Delta y_j$$

We therefore can use the inverted Riemann sum to approximate the area under the function f using the evenly spaced MC thresholds t_j as uniform subdivisions (Fig. S2D). Substituting t_j for y_j, and defining a constant Δy as the difference between each pair of thresholds:

$$Area(f) \approx \sum_{j=0}^{m-1} f^{-1}(t_j)\Delta y$$

Because $f^{-1}(t_j) = q_j' \approx q_j$:

$$Area(f) \approx \sum_{j=0}^{m-1} q_j \Delta y = \Delta y \sum_{j=0}^{m-1} q_j$$

Because $\sum_{j=0}^{m-1} \quad q_j$ is the sum of the number of MCs of all thresholds that are spiking, and is therefore equal to the total number of MCs that are spiking:

$$Q = \sum_{j=0}^{m-1} q_j$$

$$Area(f) \approx Q\Delta y$$

Because $Area(f) = c$, for constant n:

$$c \approx Q\Delta y$$

$$Q \approx \frac{c}{\Delta y}$$

$$\frac{Q}{nm} \approx \frac{c}{nm\Delta y}$$

Because the thresholds of the m MCs for each ET cell are spread evenly from 0 to u, yielding $\Delta y = \frac{u}{m}$, this finally resolves to:

$$\frac{Q}{nm} \approx \frac{c}{nu}$$

where c, n, u are all constants. That is, given the above assumptions and a constant n, c, u, the fraction of MCs that are firing approximates a constant (compare counts of *blue circles* to the left of black function f and green function g in Supplementary Fig. S4B). This finding extends to random MC thresholds drawn from a uniform distribution, with or without equivalence across columns (not shown). Using the Monte Carlo method, as the duplication factor (equivalent to m if thresholds are heterogeneous and drawn from a uniform distribution) increases, the fraction of activated MCs will approximate the fraction of the total area under the curve:

$$\frac{\#\,Firing\ MCs}{\#\,Total\ MCs} \approx \frac{Area(f)}{Sample\ Area}$$

$$\frac{Q}{nm} \approx \frac{c}{nu}$$

That is, given a sufficient duplication factor with MC thresholds drawn from a uniform distribution spanning the entire codomain of f, the fraction of activated MCs in response to normalized sensor input of any distribution will approximate a constant.

Regularization of MC Spiking Activity via Heterogeneous ET→MC Synaptic Weights

We now consider the effects of heterogeneous ET→MC synaptic weights, as presented in the main text, rather than heterogeneous MC thresholds. Here, each ET cell synapses onto m MCs exhibiting identical thresholds t, with the synaptic weights w_j from ET_i to $MC_{i,j}$ being uniformly spaced from the range $[l, u]$. $MC_{i,j}$ spikes if and only if $a_i w_j \geq t$. As above, we define the number of MCs with a given incoming weight w_j to be q_j (which will equal the number of columns). To depict the resulting generation of spikes in MCs with identical thresholds t = 8, we group all MCs by their incoming synaptic weights w_j, and plot four of these weight groups for comparison (Supplementary Fig. S4E). The synaptic weight w_j determines the ordinate scale for each plot. As above, the fraction of the horizontal threshold line to the left of the function f (Supplementary Fig. S4E, *solid red lines*) represents the number of MCs with the incoming weight w_j that are spiking.

To rescale each of the functions of Supplementary Fig. S4E onto a common ordinate scale (depicting the activation of the presynaptic ET cell), we divide each threshold t by the weight w_j for each plot to generate an *equivalent threshold* $t_j{}' = t/w_j$.

For the distribution of equivalent thresholds $t_j{}'$ to be uniform, as depicted above (Supplementary Fig. S4B), the weights w_j must be distributed as $\frac{1}{x}$ Supplementary Fig. S4F). That is, with the above assumptions, normalized input, and given a uniform MC spiking threshold t, a $\frac{1}{x}$ distribution of heterogeneous ET→MC weights ensures that the fraction of MCs that are firing approximates a constant irrespective of the input distribution.

Descriptive and Inferential Statistics

Layer	Dataset	Condition	Duplication	Accuracy Mean	Accuracy SD
GC	Concentration	Homogeneous	4	79.94047619	0.947051414
GC	Concentration	Homogeneous	8	81.59970238	1.543177004
GC	Concentration	Homogeneous	16	81.39136905	1.56257086
GC	Concentration	Homogeneous	32	81.81547619	1.097564816
GC	Concentration	Uniform	4	87.05357143	4.519219834
GC	Concentration	Uniform	8	95.53571429	2.967674892
GC	Concentration	Uniform	16	97.17261905	2.459380079
GC	Concentration	Uniform	32	97.61904762	1.558699216
GC	Concentration	Scaled	4	87.27678571	2.88003689
GC	Concentration	Scaled	8	93.45238095	2.863513306
GC	Concentration	Scaled	16	96.57738095	3.129552078
GC	Concentration	Scaled	32	97.02380952	1.683587574
GC	Concentration	Adaptive	4	90.47619048	3.883737159
GC	Concentration	Adaptive	8	92.1875	3.563447715
GC	Concentration	Adaptive	16	96.65178571	2.754268634
GC	Concentration	Adaptive	32	97.76785714	2.540368919
GC	Saturation	Homogeneous	4	56.75595238	0.972887227
GC	Saturation	Homogeneous	8	56.66666667	0.765589517
GC	Saturation	Homogeneous	16	57.3735119	1.090364369
GC	Saturation	Homogeneous	32	56.99404762	0.829681015
GC	Saturation	Uniform	4	81.77083333	4.338505874
GC	Saturation	Uniform	8	96.2797619	3.720663241
GC	Saturation	Uniform	16	99.33035714	1.250101227
GC	Saturation	Uniform	32	98.95833333	1.645578442
GC	Saturation	Scaled	4	93.82440476	4.631915198
GC	Saturation	Scaled	8	97.17261905	3.49623218
GC	Saturation	Scaled	16	98.36309524	1.413970166
GC	Saturation	Scaled	32	96.875	2.005973829
GC	Saturation	Adaptive	4	90.25297619	3.817189089
GC	Saturation	Adaptive	8	97.02380952	3.45619072
GC	Saturation	Adaptive	16	97.24702381	3.290195542
GC	Saturation	Adaptive	32	98.21428571	2.227177016
MC	Concentration	Homogeneous	4	83.75744048	0.914110702
MC	Concentration	Homogeneous	8	83.34821429	1.070365118
MC	Concentration	Homogeneous	16	83.58630952	0.937786777
MC	Concentration	Homogeneous	32	83.79464286	0.935625353
MC	Concentration	Uniform	4	87.20238095	3.149703942
MC	Concentration	Uniform	8	95.23809524	2.110488715
MC	Concentration	Uniform	16	97.09821429	2.0481109
MC	Concentration	Uniform	32	97.91666667	1.311840869
MC	Concentration	Scaled	4	88.91369048	3.05072486
MC	Concentration	Scaled	8	95.3125	1.72626378
MC	Concentration	Scaled	16	94.64285714	1.590840726
MC	Concentration	Scaled	32	95.3125	2.02324672

MC	Concentration	Adaptive	4	92.63392857	3.527758807
MC	Concentration	Adaptive	8	91.51785714	3.827947583
MC	Concentration	Adaptive	16	96.94940476	1.696689621
MC	Concentration	Adaptive	32	95.3125	2.975127361
MC	Saturation	Homogeneous	4	58.63839286	1.150448454
MC	Saturation	Homogeneous	8	58.48214286	1.365160112
MC	Saturation	Homogeneous	16	58.48214286	1.164251978
MC	Saturation	Homogeneous	32	58.66071429	1.258273559
MC	Saturation	Uniform	4	86.60714286	3.229054004
MC	Saturation	Uniform	8	92.48511905	1.620394473
MC	Saturation	Uniform	16	98.88392857	1.328613945
MC	Saturation	Uniform	32	94.56845238	5.222587395
MC	Saturation	Scaled	4	95.3125	2.34750098
MC	Saturation	Scaled	8	99.85119048	0.275541696
MC	Saturation	Scaled	16	98.51190476	1.147171561
MC	Saturation	Scaled	32	97.69345238	4.202199077
MC	Saturation	Adaptive	4	90.10416667	4.171977907
MC	Saturation	Adaptive	8	97.54464286	2.612807579
MC	Saturation	Adaptive	16	96.65178571	2.897558189
MC	Saturation	Adaptive	32	98.21428571	2.718429824

Supplementary Table S1. Analyte classification accuracy means and standard deviations as a function of spiking layer, dataset, condition, and duplication factor.

Contrast	Duplication	Layer	Estimate	SE	df	t-ratio	p-value
Homogeneous - Uniform	4	GC	-16.0639881	0.860475203	441	-18.66874031	0
Homogeneous - Scaled	4	GC	-22.20238095	0.860475203	441	-25.8024646	0
Homogeneous - Adaptive	4	GC	-22.01636905	0.860475203	441	-25.58629114	0
Uniform - Scaled	4	GC	-6.138392857	0.860475203	441	-7.133724295	2.55418E-12
Uniform - Adaptive	4	GC	-5.952380952	0.860475203	441	-6.917550831	7.57473E-11
Scaled - Adaptive	4	GC	0.186011905	0.860475203	441	0.216173463	0.996428898
Homogeneous - Uniform	8	GC	-26.77455357	0.860475203	441	-31.11600833	0
Homogeneous - Scaled	8	GC	-26.17931548	0.860475203	441	-30.42425325	0
Homogeneous - Adaptive	8	GC	-25.47247024	0.860475203	441	-29.60279409	0
Uniform - Scaled	8	GC	0.595238095	0.860475203	441	0.691755083	0.900230925
Uniform - Adaptive	8	GC	1.302083333	0.860475203	441	1.513214244	0.430396697
Scaled - Adaptive	8	GC	0.706845238	0.860475203	441	0.821459161	0.844312547
Homogeneous - Uniform	16	GC	-28.86904762	0.860475203	441	-33.55012153	0
Homogeneous - Scaled	16	GC	-28.08779762	0.860475203	441	-32.64219298	0
Homogeneous - Adaptive	16	GC	-27.56696429	0.860475203	441	-32.03690729	0
Uniform - Scaled	16	GC	0.78125	0.860475203	441	0.907928547	0.800633048
Uniform - Adaptive	16	GC	1.302083333	0.860475203	441	1.513214244	0.430396697
Scaled - Adaptive	16	GC	0.520833333	0.860475203	441	0.605285698	0.930375032
Homogeneous - Uniform	32	GC	-28.88392857	0.860475203	441	-33.56741541	0
Homogeneous - Scaled	32	GC	-27.54464286	0.860475203	441	-32.01096647	0
Homogeneous - Adaptive	32	GC	-28.58630952	0.860475203	441	-33.22153787	0
Uniform - Scaled	32	GC	1.339285714	0.860475203	441	1.556448937	0.404785457
Uniform - Adaptive	32	GC	0.297619048	0.860475203	441	0.345877542	0.98576716
Scaled - Adaptive	32	GC	-1.041666667	0.860475203	441	-1.210571395	0.62044913
Homogeneous - Uniform	4	MC	-15.70684524	0.860475203	441	-18.25368726	0
Homogeneous - Scaled	4	MC	-20.91517857	0.860475203	441	-24.30654423	0
Homogeneous - Adaptive	4	MC	-20.17113095	0.860475203	441	-23.44185038	0
Uniform - Scaled	4	MC	-5.208333333	0.860475203	441	-6.052856977	1.82241E-08
Uniform - Adaptive	4	MC	-4.464285714	0.860475203	441	-5.188163123	1.93604E-06
Scaled - Adaptive	4	MC	0.744047619	0.860475203	441	0.864693854	0.823056835
Homogeneous - Uniform	8	MC	-22.94642857	0.860475203	441	-26.66715845	0
Homogeneous - Scaled	8	MC	-26.66666667	0.860475203	441	-30.99062772	0
Homogeneous - Adaptive	8	MC	-23.61607143	0.860475203	441	-27.44538292	0
Uniform - Scaled	8	MC	-3.720238095	0.860475203	441	-4.323469269	0.000111582
Uniform - Adaptive	8	MC	-0.669642857	0.860475203	441	-0.778224469	0.864309676
Scaled - Adaptive	8	MC	3.050595238	0.860475203	441	3.545244801	0.002443481
Homogeneous - Uniform	16	MC	-26.95684524	0.860475203	441	-31.32785833	0
Homogeneous - Scaled	16	MC	-25.54315476	0.860475203	441	-29.68494	0
Homogeneous - Adaptive	16	MC	-25.76636905	0.860475203	441	-29.94434816	0
Uniform - Scaled	16	MC	1.413690476	0.860475203	441	1.642918322	0.355621578
Uniform - Adaptive	16	MC	1.19047619	0.860475203	441	1.383510166	0.510361743
Scaled - Adaptive	16	MC	-0.223214286	0.860475203	441	-0.259408156	0.993876629
Homogeneous - Uniform	32	MC	-25.01488095	0.860475203	441	-29.07100737	0
Homogeneous - Scaled	32	MC	-25.27529762	0.860475203	441	-29.37365022	0
Homogeneous - Adaptive	32	MC	-25.53571429	0.860475203	441	-29.67629307	0
Uniform - Scaled	32	MC	-0.260416667	0.860475203	441	-0.302642849	0.990364532
Uniform - Adaptive	32	MC	-0.520833333	0.860475203	441	-0.605285698	0.930375032
Scaled - Adaptive	32	MC	-0.260416667	0.860475203	441	-0.302642849	0.990364532

Supplementary Table S2. Analyte classification accuracy general linear contrast estimates, standard errors (SE), degrees of freedom (df), t-ratios, and p-values.

Model Term	df1	df2	F-ratio	p-value
Layer	1	441	5.805	0.016394082
Condition	3	441	3298.391	1.4056E-301
Duplication	3	441	179.135	6.09289E-76
Dataset	1	441	555.771	4.1112E-80
Layer:Condition	3	441	6.572	0.000235806
Layer:Duplication	3	441	5.944	0.000557259
Layer:Dataset	1	441	0.042	0.838225874
Condition:Duplication	9	441	28.307	9.38911E-39
Condition:Dataset	3	441	937.419	6.6972E-191
Duplication:Dataset	3	441	3.08	0.027316623
Layer:Condition:Duplication	9	441	1.656	0.097428339
Layer:Condition:Dataset	3	441	1.498	0.214322582
Layer:Duplication:Dataset	3	441	0.047	0.986298344
Condition:Duplication:Dataset	9	441	7.022	1.65477E-09
Layer:Condition:Duplication:Dataset	9	441	2.719	0.004295063

Supplementary Table S3. Analyte classification accuracy GLM effects, degrees of freedom, F-ratios, and p-values.

Layer	Dataset	Condition	Duplication	Regularization Mean	Regularization SD
GC	Concentration	Homogeneous	4	11.6864796	3.651219251
GC	Concentration	Homogeneous	8	11.19987165	3.23680018
GC	Concentration	Homogeneous	16	11.17477839	3.074444542
GC	Concentration	Homogeneous	32	11.25678322	3.1179467
GC	Concentration	Uniform	4	3.879449763	1.321959286
GC	Concentration	Uniform	8	3.942542236	0.916368666
GC	Concentration	Uniform	16	3.313069463	1.508523858
GC	Concentration	Uniform	32	4.555809921	1.553856109
GC	Concentration	Scaled	4	11.05426512	3.902302178
GC	Concentration	Scaled	8	6.517569274	2.27642365
GC	Concentration	Scaled	16	5.478909897	1.98343533
GC	Concentration	Scaled	32	5.263088826	1.550317777
GC	Concentration	Adaptive	4	22.77290058	6.131622727
GC	Concentration	Adaptive	8	10.27835366	4.272892392
GC	Concentration	Adaptive	16	7.201965751	1.918027423
GC	Concentration	Adaptive	32	6.400802718	1.675925721
GC	Saturation	Homogeneous	4	16.9932849	0.840534859
GC	Saturation	Homogeneous	8	16.82709804	0.710959865
GC	Saturation	Homogeneous	16	16.78360853	0.678076916
GC	Saturation	Homogeneous	32	16.74944532	0.763515635
GC	Saturation	Uniform	4	3.86770553	0.676724556
GC	Saturation	Uniform	8	3.961989606	1.071082407
GC	Saturation	Uniform	16	3.08050323	1.108177677
GC	Saturation	Uniform	32	3.013742946	1.135873255
GC	Saturation	Scaled	4	13.24707783	4.220504491
GC	Saturation	Scaled	8	7.928806939	1.765918795
GC	Saturation	Scaled	16	5.887620437	1.421716172
GC	Saturation	Scaled	32	5.315451537	1.538495658
GC	Saturation	Adaptive	4	14.12113731	7.698267568
GC	Saturation	Adaptive	8	12.15457229	4.242918981
GC	Saturation	Adaptive	16	10.5922558	3.266749874
GC	Saturation	Adaptive	32	10.05655451	2.680447304
MC	Concentration	Homogeneous	4	7.824148097	0.074457813
MC	Concentration	Homogeneous	8	7.824145469	0.074398295
MC	Concentration	Homogeneous	16	7.824148097	0.074457813
MC	Concentration	Homogeneous	32	7.824148097	0.074457813
MC	Concentration	Uniform	4	2.244967548	0.030675823
MC	Concentration	Uniform	8	0.960367407	0.02110347
MC	Concentration	Uniform	16	0.737411469	0.010411331
MC	Concentration	Uniform	32	0.59154363	0.006409456
MC	Concentration	Scaled	4	4.503799784	0.078281626
MC	Concentration	Scaled	8	1.761200722	0.062733902
MC	Concentration	Scaled	16	1.205629684	0.061771946
MC	Concentration	Scaled	32	0.933814636	0.07090784
MC	Concentration	Adaptive	4	7.719650827	0.416014399
MC	Concentration	Adaptive	8	3.551177311	0.230092824
MC	Concentration	Adaptive	16	2.301491171	0.198062336
MC	Concentration	Adaptive	32	2.065476444	0.131475482
MC	Saturation	Homogeneous	4	13.55384004	0.016201584
MC	Saturation	Homogeneous	8	13.55384004	0.016201584
MC	Saturation	Homogeneous	16	13.55384004	0.016201584
MC	Saturation	Homogeneous	32	13.55384004	0.016201584
MC	Saturation	Uniform	4	2.807856339	0.050181508
MC	Saturation	Uniform	8	1.494912709	0.040691747
MC	Saturation	Uniform	16	0.744845508	0.022611407
MC	Saturation	Uniform	32	0.447346685	0.017835972
MC	Saturation	Scaled	4	10.43286881	0.274201495
MC	Saturation	Scaled	8	4.52793637	0.123755745
MC	Saturation	Scaled	16	3.594095616	0.053574454
MC	Saturation	Scaled	32	3.062618808	0.061640575

MC	Saturation	Adaptive	4	9.898485804	1.492510947
MC	Saturation	Adaptive	8	7.949284134	0.750913344
MC	Saturation	Adaptive	16	6.361624816	0.830858088
MC	Saturation	Adaptive	32	6.127168534	0.64767331

Supplementary Table S4. Utilization regularization (standard deviation of active % of MC and GC layers) means and standard deviations as a function of spiking layer, dataset, condition, and duplication factor.

Contrast	Duplication	Layer	Estimate	SE	df	t-ratio	p-value
Homogeneous - Uniform	4	GC	10.46630461	0.716147152	441	14.61474026	0
Homogeneous - Scaled	4	GC	2.189210775	0.716147152	441	3.056928692	0.012644709
Homogeneous - Adaptive	4	GC	-4.107136693	0.716147152	441	-5.735045772	1.08456E-07
Uniform - Scaled	4	GC	-8.27709383	0.716147152	441	-11.55781157	0
Uniform - Adaptive	4	GC	-14.5734413	0.716147152	441	-20.34978603	0
Scaled - Adaptive	4	GC	-6.296347469	0.716147152	441	-8.791974464	0
Homogeneous - Uniform	8	GC	10.06121892	0.716147152	441	14.04909438	0
Homogeneous - Scaled	8	GC	6.790296736	0.716147152	441	9.48170599	0
Homogeneous - Adaptive	8	GC	2.797021865	0.716147152	441	3.905652433	0.000626949
Uniform - Scaled	8	GC	-3.270922185	0.716147152	441	-4.56738839	3.79243E-05
Uniform - Adaptive	8	GC	-7.264197057	0.716147152	441	-10.14344195	0
Scaled - Adaptive	8	GC	-3.993274871	0.716147152	441	-5.576053557	2.5693E-07
Homogeneous - Uniform	16	GC	10.78240712	0.716147152	441	15.0561335	0
Homogeneous - Scaled	16	GC	8.295928297	0.716147152	441	11.58411128	0
Homogeneous - Adaptive	16	GC	5.082082691	0.716147152	441	7.096422404	9.19897E-12
Uniform - Scaled	16	GC	-2.48647882	0.716147152	441	-3.472022216	0.00317331
Uniform - Adaptive	16	GC	-5.700324426	0.716147152	441	-7.959711093	0
Scaled - Adaptive	16	GC	-3.213845606	0.716147152	441	-4.487688877	5.42657E-05
Homogeneous - Uniform	32	GC	10.21833783	0.716147152	441	14.26848911	0
Homogeneous - Scaled	32	GC	8.713844085	0.716147152	441	12.16767262	0
Homogeneous - Adaptive	32	GC	5.774435652	0.716147152	441	8.063197123	0
Uniform - Scaled	32	GC	-1.504493748	0.716147152	441	-2.100816494	0.1544181
Uniform - Adaptive	32	GC	-4.443902181	0.716147152	441	-6.205291988	7.52837E-09
Scaled - Adaptive	32	GC	-2.939408433	0.716147152	441	-4.104475494	0.000281197
Homogeneous - Uniform	4	MC	8.162582124	0.716147152	441	11.39791188	0
Homogeneous - Scaled	4	MC	3.220659771	0.716147152	441	4.497203912	5.20084E-05
Homogeneous - Adaptive	4	MC	1.879925753	0.716147152	441	2.625055128	0.044243049
Uniform - Scaled	4	MC	-4.941922353	0.716147152	441	-6.900707965	8.67574E-11
Uniform - Adaptive	4	MC	-6.282656371	0.716147152	441	-8.772856749	0
Scaled - Adaptive	4	MC	-1.340734019	0.716147152	441	-1.872148783	0.24157166
Homogeneous - Uniform	8	MC	9.461352695	0.716147152	441	13.21146454	0
Homogeneous - Scaled	8	MC	7.544424208	0.716147152	441	10.53474023	0
Homogeneous - Adaptive	8	MC	4.938762031	0.716147152	441	6.896295015	8.98387E-11
Uniform - Scaled	8	MC	-1.916928488	0.716147152	441	-2.676724307	0.03848173
Uniform - Adaptive	8	MC	-4.522590664	0.716147152	441	-6.315169522	3.93219E-09
Scaled - Adaptive	8	MC	-2.605662176	0.716147152	441	-3.638445215	0.001739077
Homogeneous - Uniform	16	MC	9.947865579	0.716147152	441	13.89081219	0
Homogeneous - Scaled	16	MC	8.289131418	0.716147152	441	11.57462038	0
Homogeneous - Adaptive	16	MC	6.357436074	0.716147152	441	8.877276214	0
Uniform - Scaled	16	MC	-1.658734161	0.716147152	441	-2.316191802	0.095888886
Uniform - Adaptive	16	MC	-3.590429505	0.716147152	441	-5.013535971	4.61822E-06
Scaled - Adaptive	16	MC	-1.931695344	0.716147152	441	-2.697344169	0.036368778
Homogeneous - Uniform	32	MC	10.16954891	0.716147152	441	14.20036216	0
Homogeneous - Scaled	32	MC	8.690777346	0.716147152	441	12.13546312	0
Homogeneous - Adaptive	32	MC	6.592671578	0.716147152	441	9.205749914	0
Uniform - Scaled	32	MC	-1.478771565	0.716147152	441	-2.064899039	0.166330192
Uniform - Adaptive	32	MC	-3.576877332	0.716147152	441	-4.994612245	5.0668E-06
Scaled - Adaptive	32	MC	-2.098105767	0.716147152	441	-2.929713206	0.018658942

Supplementary Table S5. Utilization regularization (standard deviation of active % of MC and GC layers) general linear contrast estimates, standard errors (SE), degrees of freedom (df), t-ratios, and p-values.

Model Term	df1	df2	F-ratio	p-value
Layer	1	441	476.183	3.96306E-72
Condition	3	441	559.527	6.8404E-150
Duplication	3	441	91.858	3.40888E-46
Dataset	1	441	177.808	2.56168E-34
Layer:Condition	3	441	17.085	1.63219E-10

Layer:Duplication	3	441	3.314	0.019955889
Layer:Dataset	1	441	22.519	2.81649E-06
Condition:Duplication	9	441	21.523	3.1216E-30
Condition:Dataset	3	441	44.078	6.25657E-25
Duplication:Dataset	3	441	2.041	0.107374497
Layer:Condition:Duplication	9	441	3.765	0.000138808
Layer:Condition:Dataset	3	441	4.683	0.00312121
Layer:Duplication:Dataset	3	441	4.212	0.005929607
Condition:Duplication:Dataset	9	441	8.261	2.14031E-11
Layer:Condition:Duplication:Dataset	9	441	2.721	0.004274631

Supplementary Table S6. Utilization regularization (standard deviation of active % of MC and GC layers) GLM effects, degrees of freedom, F-ratios, and p-values.
